# Defect Engineering and Carbon Supporting to Achieve Ni-Doped CoP_3_ with High Catalytic Activities for Overall Water Splitting

**DOI:** 10.1007/s40820-024-01471-9

**Published:** 2024-07-18

**Authors:** Daowei Zha, Ruoxing Wang, Shijun Tian, Zhong-Jie Jiang, Zejun Xu, Chu Qin, Xiaoning Tian, Zhongqing Jiang

**Affiliations:** 1 Department of Physics, Zhejiang Sci-Tech University, Hangzhou, 310018 People’s Republic of China; 2 Department of Materials and Chemical Engineering, Ningbo University of Technology, Ningbo, 315211 People’s Republic of China; 3 Guangzhou Key Laboratory for Surface Chemistry of Energy Materials, Guangdong Engineering and Technology Research Center for Surface Chemistry of Energy Materials, College of Environment and Energy, South China University of Technology, Guangzhou, 510006 People’s Republic of China

**Keywords:** Plasma, Electrocatalysis, Hydrogen evolution reaction, Oxygen evolution reaction, Water splitting

## Abstract

This work reports the use of defect engineering and carbon supporting to achieve metal-doped phosphides with high activities and stabilities for the hydrogen evolution reaction (HER) and the oxygen evolution reaction (OER) in alkaline media. Specifically, the nitrogen-doped carbon nanofiber-supported Ni-doped CoP_3_ with rich P defects (Pv·) on the carbon cloth (p-NiCoP/NCFs@CC) is synthesized through a plasma-assisted phosphorization method. The p-NiCoP/NCFs@CC is an efficient and stable catalyst for the HER and the OER. It only needs overpotentials of 107 and 306 mV to drive 100 mA cm^−2^ for the HER and the OER, respectively. Its catalytic activities are higher than those of other catalysts reported recently. The high activities of the p-NiCoP/NCFs@CC mainly arise from its peculiar structural features. The density functional theory calculation indicates that the Pv· richness, the Ni doping, and the carbon supporting can optimize the adsorption of the H atoms at the catalyst surface and promote the strong electronic couplings between the carbon nanofiber-supported p-NiCoP with the surface oxide layer formed during the OER process. This gives the p-NiCoP/NCFs@CC with the high activities for the HER and the OER. When used in alkaline water electrolyzers, the p-NiCoP/NCFs@CC shows the superior activity and excellent stability for overall water splitting.

**Graphical abstract:**

**Supplementary Information:**

The online version contains supplementary material available at 10.1007/s40820-024-01471-9.

## Highlights

- Plasma-assisted phosphorization has been used to prepare defect-rich metal phosphides.
- The p-NiCoP/NCF@CC shows high activities and excellent stability for the hydrogen evolution reaction and the oxygen evolution reaction.
- The p-NiCoP/NCF@CC shows great potential for overall water splitting.

**Supplementary Information:**

The online version contains supplementary material available at 10.1007/s40820-024-01471-9.

## Graphical abstract

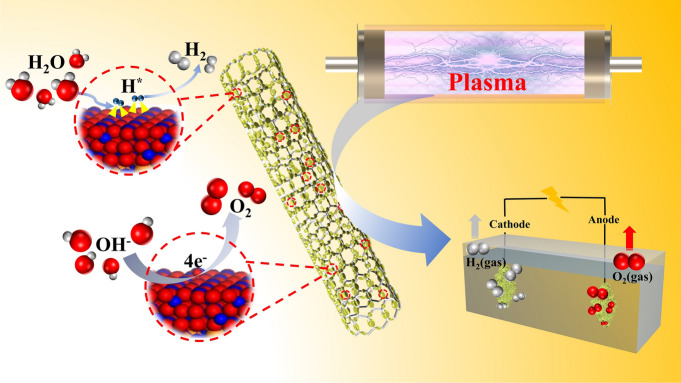

## Introduction

With continuous growth of population and increasing demand of energy, exploiting alternative energy sources to replace tradition fossil fuels is of great importance [1–3]. Hydrogen (H_2_), as a clean and sustainable energy carrier, has shown great promises to replace traditional fossil fuels because of its zero carbon emissions and high energy density [4–6]. Hydrogen production through electrochemical water splitting has been well recognized as an ideal and eco-friendly approach for hydrogen generation [7, 8]. Specifically, to achieve the water splitting with high efficiencies, the electrocatalysts with high activities and high stabilities are compulsorily required to drive anodic oxygen evolution reaction (OER) and cathodic hydrogen evolution reaction (HER), which are two important reactions associated with electrochemical water electrolyzers [9, 10]. Currently, both the HER and the OER use noble metal-based nanoparticles (e.g., Pt, Ru, Ir) and/or their derivatives as the electrocatalysts. The low reserves and high cost of these precious metal have, however, greatly reduced the practical usability of the electrochemical water splitting [11, 12]. The development of inexpensive catalysts with excellent catalytic activities and stabilities, especially those with the bifunctional HER/OER activities, is therefore highly desirable [13, 14]. The inexpensive bifunctional catalysts allow to simultaneously boost the kinetics of the OER and the HER to give the electrochemical water splitting with high efficiencies and low costs [15–17].

Among various materials reported to date, transition metal phosphides (TMPs) have shown great promises due to their capabilities to simultaneously drive the HER and the OER [18]. However, to make them competitive with traditional noble metal-based catalysts, the catalytic activities and stabilities of these TMP-based catalysts still need to be increased substantially [19]. Generally, the activity of a catalyst is greatly determined by its surface electronic structure, since it influences the reactant adsorption, the intermediate transformation, and the product release during the catalytic processes [20, 21]. Various approaches, including carbon supporting, defect engineering, elemental doping, etc., have, therefore, been explored to the optimization of the surface electronic structures of the TMP-based catalysts to give them with optimal catalytic activities. For example, the carbon supporting can lead to a strong electronic coupling between the TMPs and the carbon supports. It cannot only regulate the surface electronic structure of the TMP through the charge redistribution to give the TMPs with high HER and OER activities, but also increases the stabilities of the catalysts by reducing the surface energy of the TMPs through the strong catalyst-support interactions. Additionally, the high electric conductivity of the carbon supports can also suppress the activity loss of the catalysts caused by the resistance induced electrochemical polarization [22, 23]. The defect engineering and elemental doping can modulate the metal-phorsphorous covalency in the TMPs, optimizing the intermediate adsorption and increasing the intrinsic catalytic activities and stabilities of the TMP-based catalysts [24]. Great interest is that the multiple approaches can be combined to cooperatively increase the activities and stabilities of the TMP-based catalysts toward the HER and the OER. In this way, the advantages of various approaches are expected to be integrated. This, however, remains a great challenge due to the difficults in the lack of the reliable chemical approaches to directly prepare the catalysts with multiple advanced features.

This work reports the use of a plasma method to prepare carbon-supported Ni-doped Co phosphides with phosphorus defects (Pv·). Specifically, nitrogen-doped carbon nanofiber (NCF)-supported Ni-doped CoP_3_ nanoparticles (NPs) with rich Pv· on carbon cloth (p-NiCoP/NCFs@CC) have been synthesized through the plasma-assisted phosphorization of the NiCoLDH-deposited NCFs@CC in the presence of NaH_2_PO_2_. The strategy of this work is to improve the HER and OER activities and stabilities of metal-doped phosphides through the defect engineering and carbon supporting. Interestingly, due to the advanced structure features, which integrates the carbon supporting, defect engineering and metal doping, the p-NiCoP/NCFs@CC exhibits superior bifunctional activities and stabilities for the HER and the OER in the alkaline media. It only needs overpotentials of 107 and 306 mV to drive 100 mA cm^−2^ for the HER and the OER, respectively. The Pv· richness, the carbon fibers supporting, and the Ni doping have shown to be the main origins of the high OER and HER activities of the p-NiCoP/NCFs@CC. The density functional theory (DFT) calculation indicates that the Pv· richness, the Ni doping, and the carbon fibers supporting optimize the adsorption of the H atoms at the catalyst surface and promote the strong electronic couplings between the carbon fibers-supported p-NiCoP NPs with the surface oxide layer formed during the OER process. This gives the p-NiCoP/NCFs@CC with the high activity for the HER and the OER. More impressively, the alkaline water electrolyzers assembled with the p-NiCoP/NCFs@CC as both the anode and cathode catalyst exhibit the superior activity and excellent stability for overall water splitting.

## Experimental Section

### Material Synthesis

#### Synthesis of NCFs@CC

The carbon cloth (CC, 3 cm × 4 cm) was first immersed in a 10 wt% potassium permanganate solution under the sonication for 10 min and then thoroughly washed with deionized (DI) water and ethanol. The cleaned CC was then placed in a Teflon-lined stainless steel autoclave (50 mL) and 40 mL water containing 0.39 g Co (NO_3_)_2_·6H_2_O, 0.27 g Fe (NO_3_)_3_·9H_2_O, 0.18 g NH_4_F and 0.60 g urea was then poured in. The reaction system was subsequently hydrothermally heat at 120 °C for 6 h. After cooling, the CC was taken out, washed with water and ethanol in turn, and dried in an oven at 40 °C. The dried CC and 1.5 g of dicyandiamide (DCDA) were separately loaded in two different porcelain boats and placed into a tube furnace. Specifically, the DCDA-loaded porcelain boat was placed in the upstream of the tube furnace. The tube furnish was then heated at 400 °C for 2 h and 750 °C for 90 min, respectively, at a heating rate of 5 °C min^−1^. This led to the formation of the NCFs@CC.

#### Synthesis of NiCoLDH/NCFs@CC

The NiCoLDH/NCFs@CC was prepared by a hydrothermal method. Typically, 0.95 g NiCl_2_·6H_2_O, 1.90 g CoCl_2_·6H_2_O and 1.50 g urea were dissolved in 70 mL DI water under stirring. The obtained homogeneous solution was then poured into a Teflon-lined stainless steel autoclave (100 mL) placed with the NCFs@CC. The reaction system was then hydrothermally heated at 120 °C for 6 h. After cooling, the obtained NiCoLDH/NCFs@CC was successively washed with ethanol and DI water and dried at 60 °C.

#### Plasma-Assisted Phosphating of p-NiCoP/NCFs@CC

The p-NiCoP/NCFs@CC was obtained through a plasma-assisted phosphating approach. Specifically, the NiCoLDH/NCFs@CC synthesized above was placed in the downstream side in a porcelain boat and was calcined in the oven at 300 °C for 90 min in N_2_ atmosphere in the presence of 1.5 g NaH_2_PO_2_ on the upstream side. The radio-frequency (RF) plasma discharge was then triggered with the flow of N_2_ (5 sccm) at the pressure of 20 Pa. The RF plasma discharge power was set at 100 W. The mass loading of phosphide was estimated to be 18 mg cm^−2^ after the RF plasma-assisted phosphating reaction. For comparison, the NiCoP/NCFs@CC was obtained by the direct thermal calcination under the same conditions without RF plasma discharge. The p-CoP/NCFs@CC and p-NiP/NCFs@CC with single metal and the p-NiCoP@CC deposited directly on the carbon cloth were also synthesized for comparison.

### Characterizations

The structures of the samples are characterized by high-resolution field emission scanning electron microscope (SEM) (FEI, Verios G4) and high-resolution transmission electron microscope (TEM) (FEI, Talos F200S). X-ray diffraction (XRD) patterns were recorded on an X-ray diffractometer at 40 kV using a Cu Kα irradiation source (λ = 1.54 Å). Brunauer–Emmett–Teller (BET) surface area of the sample was examined on an automatic volumetric sorption analyzer (Quantachrome, Autosorb-IQ-MP). Raman spectra were obtained using a Lab RAM HR Evolution spectrometer (Jobin–Yvon HR 800). Electron paramagnetic resonance (EPR) spectra were collected on a JES-FA200 spectrometer. X-ray photoelectron spectra (XPS) were obtained on a Thermo VG Scientific ESCALAB 250 with using the Al Kα as the radiation source.

## Result and Discussion

### Structural Characterization

**Fig. 1:**
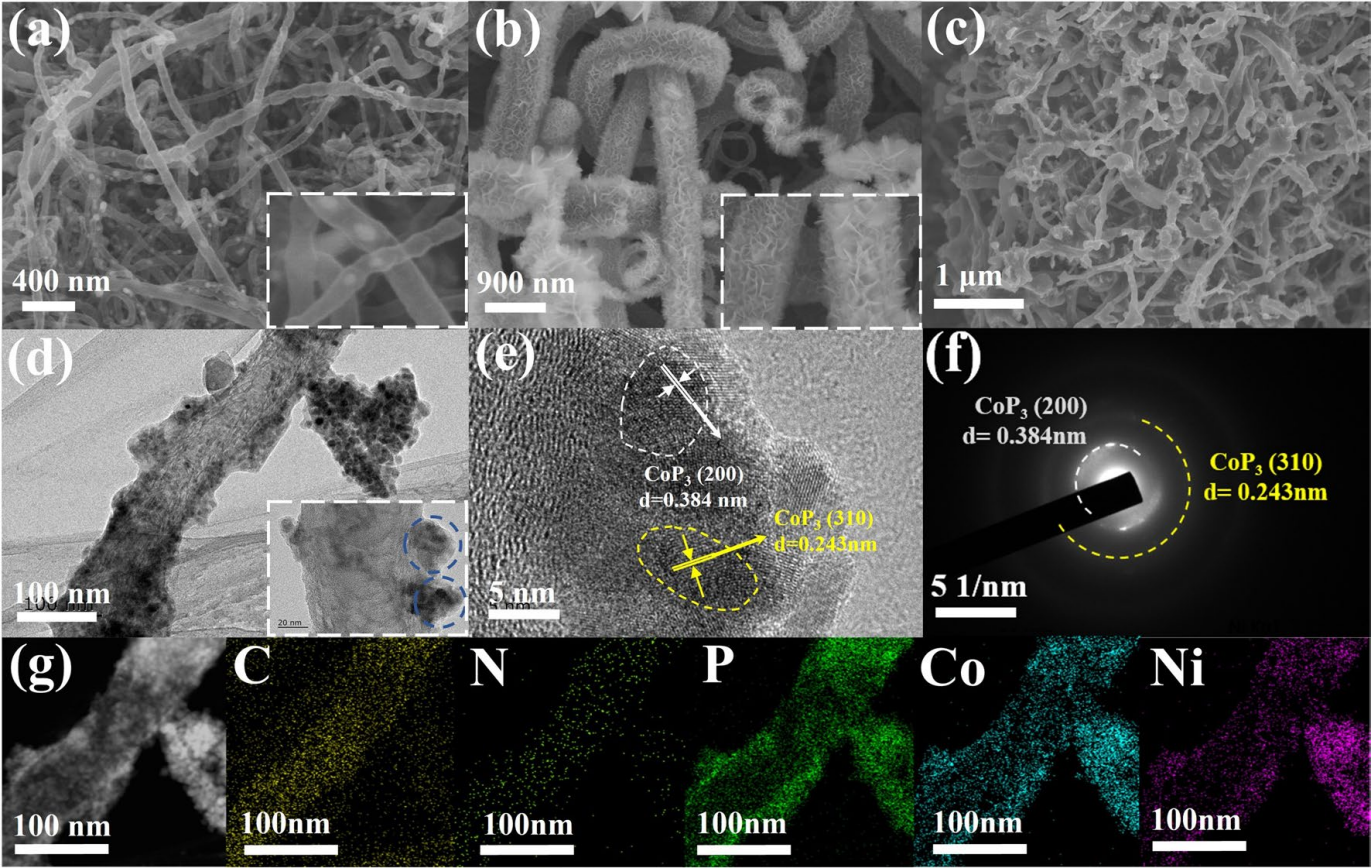
SEM images of **a** NCFs@CC, **b** NiCoLDH/NCFs@CC, and **c** p-NiCoP/NCFs@CC; **d** TEM, **e** HR-TEM, **f** SAED pattern, and **g** corresponding EDX elemental mapping images of p-NiCoP/NCFs scraped form the p-NiCoP/NCFs@CC

The p-NiCoP/NCFs@CC is synthesized following a three-step procedure as shown in Fig. S1. Specifically, the NCFs@CC is first prepared by the growth NCFs on the CC through the calcination of the FeCo LDH deposited CC in the presence of dicyandiamide (step I). NiCo LDH is then deposited onto the NCFs@CC through the hydrothermal reaction of NiCl_2_, CoCl_2_ and urea in the presence of the NCFs@CC (step II). The formation of the p-NiCoP/NCFs@CC is finally achieved by the plasma-assisted phosphorization of the NiCo LDH deposited NCFs@CC (NiCoLDH/NCFs@CC) in the presence of NaH_2_PO_2_ (step III). Figure S2a shows that the pristine CC has a smooth surface. The growth of the NCFs on the CC can be well evidenced by the SEM image of the NCFs@CC in Figs. 1a and S2d. These NCFs have a tube-like structure, in which the encapsulation of small-sized nanoparticles (NPs) in the tubes can be visualized (Fig. S2e). HRTEM indicates that the small sized NPs are metallic Co or CoFe. As shown in Fig. S2f, the lattice fringes corresponding to the (111) and (110) planes of the cubic structure Co (d = 0.205 nm) and CoFe (d = 0.201 nm) can be clearly observed. These Co and CoFe NPs are formed from the FeCo LDH deposited on the surface of the CC during the high-temperature calcination. They can work as the catalysts, facilitating the formation of the NCFs. This can be well evidenced by Fig. S2b, c, which shows the absence of NCFs growth when the CC without the deposition of the FeCo LDH is directly calcined in the presence of dicyandiamide. Except for those corresponding to Co(111) and CoFe(110), the HRTEM image of the NCFs also shows the lattice fringes assignable to the (002) plane of the graphitic carbon. It validates the formation of the NCFs.

The formation of the NiCoLDH/NCFs@CC is accomplished by the growth of the NiCo LDH on the NCFs@CC through a hydrothermal reaction. Figure S2g shows that the NiCoLDH/NCFs@CC has a rough surface. NiCo LDH on the surface of the NCFs@CC has a sheet-like structure, as revealed by the high resolution SEM image in Fig. 1b. Analysis by the XRD pattern reveals that NiCo LDH consists of mixed phases of Co(CO_3_)_0.5_(OH)·0.11H_2_O and Ni_6.10_Co_2.90_(OH)_18.27_(CO_3_)_1.315_·6.7H_2_O. As shown in Fig. S3, the diffraction peaks corresponding to the (020), (221), and (231) reflections of the orthorhombic structure Co(CO_3_)_0.5_(OH)·0.11H_2_O (JCPDS No. 48-0083) and corresponding to the (003), (006), (012), and (015) reflections of the rhombic structure Ni_6.10_Co_2.90_(OH)_18.27_(CO_3_)_1.315_·6.7H_2_O (JCPDS No. 33-0429) can be observed in the XRD pattern of the NiCoLDH/NCFs@CC.

**Fig. 2:**
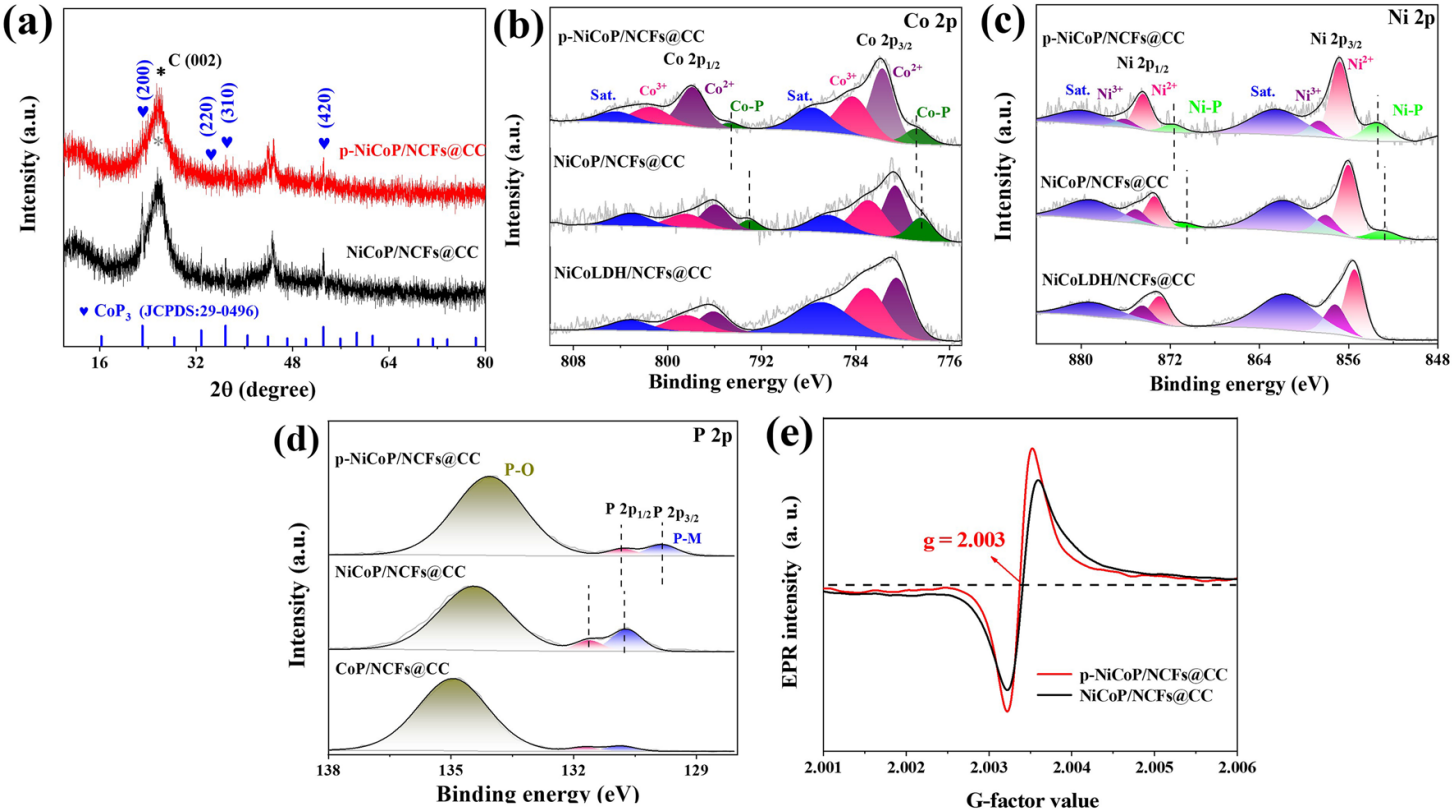
**a** XRD patterns of p-NiCoP/NCFs@CC and NiCoP/NCFs@CC. XPS spectra of **b** Co 2*p*, **c** Ni 2*p* and **d** P 2*p* for the p-NiCoP/NCFs. **e** EPR spectra

The plasma treatment of the NiCoLDH/NCFs@CC in the presence of NaH_2_PO_2_ at the high temperature will lead to the phosphorization of NiCo LDH. SEM reveals that the p-NiCoP/NCFs@CC exhibits a morphology consisting of intertwined nanowires with no sheet-like materials observed. It suggests the morphology evolution of the NiCo LDH nanosheets during the plasma-assisted phosphorization. Figure 1d gives a typical TEM image of a nanowire scraped from the p-NiCoP/NCFs@CC. It discloses that the nanowire is composed of the NCFs-supported p-NiCoP NPs. The p-NiCoP NPs are crystallized with the lattice fringes clearly observable (Fig. 1e). The distances measured from the lattice fringes are 0.384 and 0. 243 nm, well close to the d-spacings of the (200) and (310) planes of the cubic structured CoP_3_, respectively. It indicates that these p-NiCoP NPs are Ni-doped CoP_3_. This can be well demonstrated by the selected area electron diffraction (SAED) image in Fig. 1f, which shows the diffraction rings corresponding to the (200) and (310) planes of the cubic structure CoP_3_. Figure 2a displays the XRD pattern of the p-NiCoP/NCFs, which shows the diffraction peaks at 23.05°, 32.83°, 36.86°, and 53.10°, indexable to the (200), (220), (310), and (420) reflections of the cubic structured CoP_3_ (PDF No. 29-0496). It further demonstrates the presence of the p-NiCoP NPs in the p-NiCoP/NCFs. Except for the peaks corresponding to CoP_3_, the XRD pattern of the p-NiCoP/NCFs exhibits a broad peak at 26.50°, which can be ascribed to the diffraction of the (002) plane of the graphitic carbon. The absence of the peaks corresponding to Co(CO_3_)_0.5_(OH)·0.11H_2_O and Ni_6.10_Co_2.90_(OH)_18.27_(CO_3_)_1.315_·6.7H_2_O implies the full transformation of NiCo LDH into p-NiCoP NPs. In particular, to give an additional support that the p-NiCoP/NCFs has a structure which consists of the NCFs-supported p-NiCoP NPs, the EDX elemental mapping images are taken. Figure 1g shows that the p-NiCoP/NCFs is mainly composed of Co, Ni, C, N, and P, which distribute along the longitudinal direction of the NCFs.

The XPS spectroscopic measurements further evidence that the p-NiCoP/NCFs is composed of Co, Ni, C, N, and P (Fig. S4a). The high-resolution Co 2*p* spectrum shows several distinct peaks (Fig. 2b). The peak at 794.74 and 778.83 eV can be assigned to the respective Co 2*p*_1/2_ and Co 2*p*_3/2_ of Co bonded to P (Co-P), the peaks at 801.44/798.87 and 784.24/781.72 eV can be assigned to the respective Co 2*p*_1/2_ and Co 2*p*_3/2_ of Co^3+^/Co^2+^, while the peaks at 804.32 and 787.64 eV are assignable to the satellite peaks of Co 2*p*_1/2_ and Co 2*p*_3/2_, respectively. The appearance of the peaks corresponding to Co-P suggests the presence of the phosphides in the p-NiCoP/NCFs@CC. The surface of the phosphides is readily oxidized due to the exposure to air, as reported previously [25, 26]. The emergence of Co at the high oxidation states of + 2/+ 3 well evidences the partial surface oxidation of the phosphides in the p-NiCoP/NCFs. Similar to Co 2*p*, the Ni 2*p* spectrum also shows the peaks corresponding to Ni–P and Ni^3+/2+^ and the satellite peaks of Ni 2p_1/2_ and Ni 2p_3/2_, respectively (Fig. 2c). Figure 2d gives the P 2*p* spectrum of the p-NiCoP/NCFs. Spectra deconvolution shows the presence of the peaks at 130.80 and 129.83 eV, corresponding to P 2*p*_1/2_ and P 2*p*_3/2_ of P bonded to M (P-M). The broad peak at 134.06 eV is attributable to P bonded to O (P-O). These results further demonstrate the presence of the phosphides in the p-NiCoP/NCFs with the surface partially oxidized. The C 1*s* spectrum indicates that the p-NiCoP/NCFs are dominated with *sp*^2^-hybridized carbon, which gives the p-NiCoP/NCFs with high electric conductivity (Fig. S4b). The presence of the peaks corresponding to C-N indicates that the p-NiCoP/NCFs have a nitrogen-doped structure. This can be further demonstrated by the N 1*s* spectrum in Fig. S4c, which shows the peaks corresponding to the graphitic, pyrrolic, and pyridinic N, respectively. The atomic percentage of N in the p-NiCoP/NCFs is low (atomic at 0.56%), as demonstrated by the XPS analysis.

**Fig. 3:**
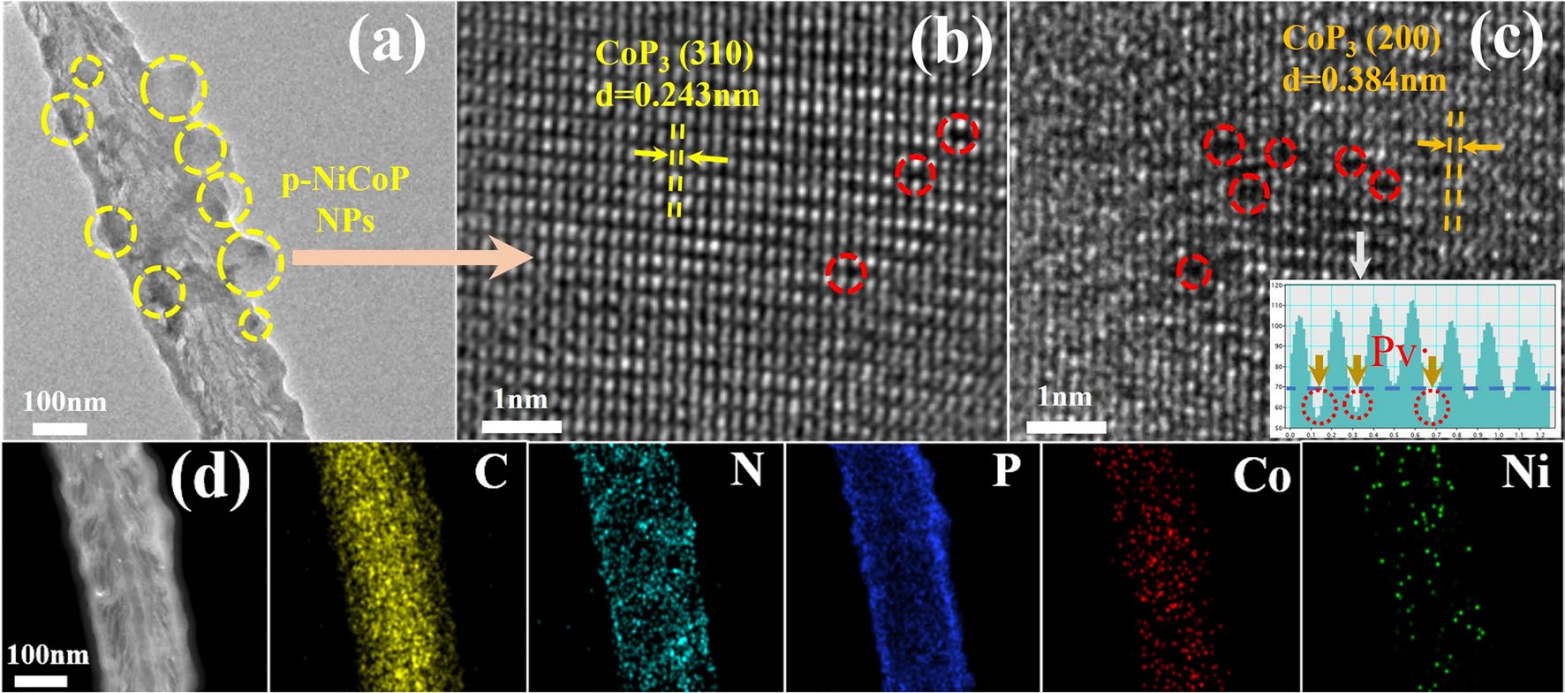
**a** AC-STEM image of p-NiCoP/NCFs. **b**, **c** AC-STEM image of the single p-NiCoP NPs. **d** High-resolution elemental mapping images of p-NiCoP/NCFs

The p-NiCoP NPs in the p-NiCoP/NCFs@CC are rich with P vacancies (Pv·). As shown by Fig. 2e, the distinct peak at g = 2.003, corresponding to Pv·, can be clearly observed in the electron paramagnetic resonance (EPR) spectra of the p-NiCoP/NCFs. The Pv·-rich structure of the p-NiCoP NPs can be further demonstrated by the spherical aberration-corrected scanning transmission electron microscope (AC-STEM) image of the p-NiCoP/NCFs. Consistent with the TEM results, the AC-STEM image of the p-NiCoP/NCFs exhibits a structure consisting of the NCFs with the growth of the p-NiCoP NPs (Fig. 3a). The lattice fringes corresponding to the (200) and (310) faces of the cubic structure CoP_3_ can be clearly observed in the AC-STEM image of the single p-NiCoP NPs (Fig. 3b, c). Closer inspection shows the existence of discontinuity and metal ion dislocations in the lattice fringes of the p-NiCoP NPs. This is verified by the intensity line projection, which shows the variations in the intensity and distance of the atomic projections (Fig. 3c, inset). In particular, the Pv·-rich structure of the p-NiCoP NPs can be further demonstrated by the ICP-OES analysis, which shows that the atomic ratio of Co:Ni:P in the p-NiCoP@NCNTs is 1:0.47:3.03. It is under the stoichiometry of Co(Ni)P_3_. The weight percentage of p-NiCoP in the p-NiCoP/NCFs@CC is estimated to be 55.23% by weighing the masses of the p-NiCoP/NCFs@CC before and after the p-NiCoP removal. Due to the specific structure which consists of the CC-supported NCFs with the growth of p-NiCoP NPs, the p-NiCoP/NCFs@CC exhibits a high BET specific surface area of 44.4 m^2^ g^−1^, as shown by the N_2_ adsorption–desorption isotherms in Fig. S5.

Control experiments indicate that the growth of Ni-doped CoP_3_ NPs on the NCFs@CC (NiCoP/NCFs@CC) can also be achieved through the thermal calcination of the NiCoLDH/NCFs@CC in the absence of the plasma treatment. The NiCoP/NCFs@CC shows the structure comparable to the p-NiCoP/NCFs@CC (Fig. S6). The NiCoP/NCFs@CC is, however, shown to have a relatively lower BET specific area (20.6 m^2^ g^−1^, Fig. S5). It indicates that the plasma-assisted phosphorization is conducive to the exposure of the p-NiCoP NPs to give more active sites accessible to the catalytic reactions. In particular, the EPR spectra indicate that the NiCoP/NCFs@CC synthesized by the conventional thermal calcination exhibits relatively lower signals corresponding to Pv· (Fig. 2e), indicative of a less defective structure of the NiCoP NPs in the NiCoP/NCFs@CC. The presence of more Pv· in the p-NiCoP NPs of the p-NiCoP/NCFs@CC is the common structural feature of the materials fabricated by the plasma-assisted method. As reported previously, the plasma fabrication method is one of the most promising techniques to manufacture defective materials [27]. It relies on highly energetic species (such as gaseous radicals, ions, atoms, and molecules) generated during the plasma discharge to prepare or modify the materials to give them with peculiar structural features. Specifically, the presence of more Pv· in the p-NiCoP NPs can be well demonstrated by the XPS spectra of Co 2*p*, Ni 2*p*, and P 2*p* in Fig. 2b–d. As shown in Fig. 2b–d, although the p-NiCoP/NCFs@CC exhibits the Co 2*p*, Ni 2*p*, and P 2*p* spectra with profiles comparable to the NiCoP/NCFs@CC, the peaks corresponding to Co–P/Ni–P at the higher binding energies and the peaks corresponding to P-M at the lower binding energies can be observed in the Co 2*p*/Ni 2*p* and P 2*p* spectra, respectively. The Pv·-rich structure makes each P atom accept more charges from Co and Ni.

### HER and OER Activities

**Fig. 4:**
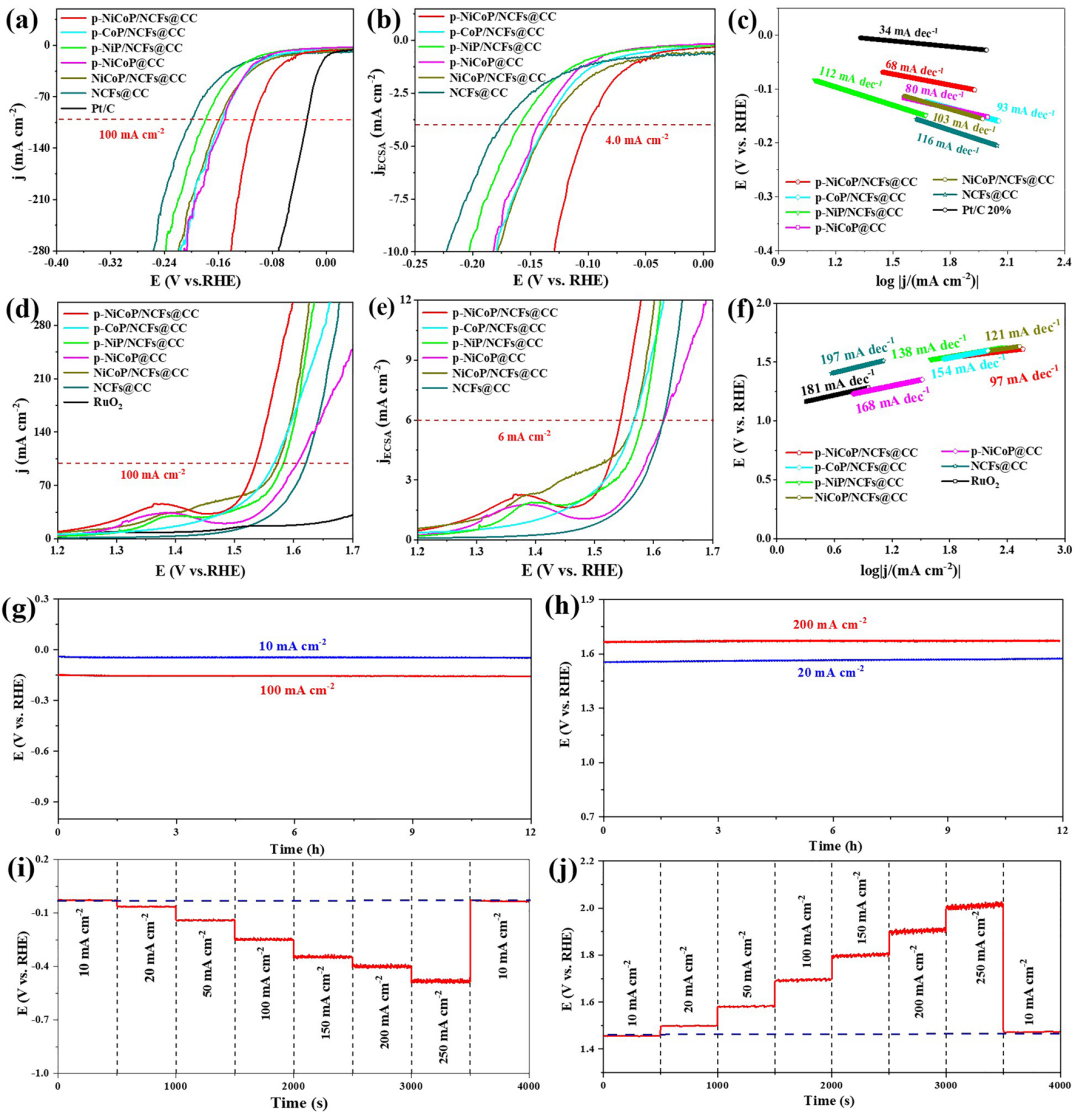
**a** Polarization curves, **b** ECSA-normalized polarization curves, and **c** Tafel curves for the HER, **d** Polarization curves, **e** ECSA-normalized polarization curves, **f** Tafel curves for the OER. Chronopotentiometric curves of the p-NiCoP/NCFs@CC for **g** HER and for **h** OER. Multi-step chronopotentiometric curves of the p-NiCoP/NCFs@CC for **i** HER and **j** OER

The p-NiCoP/NCFs@CC is an efficient catalyst for the HER in the alkaline media (1.0 M KOH). The polarization curves in Fig. 4a indicate that the p-NiCoP/NCFs@CC only needs an overpotential of 107 mV to drive 100 mA cm^−2^ ($\eta_{{{\text{HER}}.100}}$ = 107 mV). This overpotential is much lower than that of the NiCoP/NCFs@CC ($\eta_{{{\text{HER}}.100}}$ = 159 mV). It suggests that the plasma-assisted phosphorization, which gives the p-NiCoP/NCFs@CC with the higher BET specific surface area and the Pv·-rich structure, can give the p-NiCoP/NCFs@CC with the high activity for the HER. The active sites of the p-NiCoP/NCFs@CC for the HER are on the p-NiCoP NPs. This can be demonstrated by the observation that both the CC (Fig. S7) and NCFs@CC ($\eta_{{{\text{HER}}.100}}$ = 200 mV) in the absence of the p-NiCoP NPs exhibits extremely lower activities for the HER. The bimetallic structure of the p-NiCoP NPs is confirmed to be an important factor leading to the high activity of the p-NiCoP/NCFs@CC. As shown in Fig. 4a, both the p-CoP/NCFs@CC ($\eta_{{{\text{HER}}.100}}$ = 155 mV) and the p-NiP/NCFs@CC ($\eta_{{{\text{HER}}.100}}$ = 178 mV) with the single metal phosphides synthesized by the same method show much lower HER activities than the p-NiCoP/NCFs@CC. Figure 4a shows that the p-NiCoP@CC ($\eta_{{{\text{HER}}.100}}$ = 151 mV) synthesized in the absence of the NCFs exhibits a lower HER activity than the p-NiCoP/NCFs@CC. It suggests that the NCFs are indispensable in the high catalytic activity of the p-NiCoP/NCFs@CC. These NCFs can, on one hand, give a substrate for the growth of the p-NiCoP NPs, preventing the aggregation of the p-NiCoP NPs and promoting the exposure of more active sites to the catalytic reactions. Their high electric conductivity, on the other hand, can facilitate the electron transfer during the catalytic reactions, reducing the polarization losses of energy caused by the low resistance of the phosphide-based catalysts. In particular, although the p-NiCoP/NCFs@CC exhibits the lower HER activity than the commercial Pt/C ($\eta_{{{\text{HER}}.100}}$ = 29 mV, Fig. 4a), the HER activity of the p-NiCoP/NCFs@CC in the alkaline media is higher than the most non-noble metal-based catalysts reported recently, such as MnCoP/CC [28], NiFeCuP [29], and CeO_2_-NiCoP_x_/NCF [30] (Table S1).

The intrinsic HER activities of the catalysts are evaluated by the electrochemical active surface area (ECSA)-normalized polarization curves. Specifically, the ECSA of the catalyst is estimated from its double-layer capacitance (*C*_dl_), which is obtained through measuring its CVs in the non-Faradiac range at the different sweeping rates (Fig. S8). The ECSA-normalized polarization curves in Fig. 4b shows that the p-NiCoP/NCFs@CC only needs an overpotential of 101 mV to drive 4 mA cm^−2^ (${\eta }_{HER,4}^{ECSA}$= 101 mV), which is lower than those of NiCoP/NCFs@CC (${\eta }_{HER,4}^{ECSA}$= 136 mV), p-NiCoP@CC (${\eta }_{HER,4}^{ECSA}$= 144 mV), p-CoP/NCFs@CC (${\eta }_{HER,4}^{ECSA}$= 137 mV), p-NiP/NCFs@CC(${\eta }_{HER,4}^{ECSA}$ = 159 mV), and NCFs@CC (${\eta }_{HER,4}^{ECSA}$ = 175 mV). It indicates that the HER activity of the p-NiCoP/NCFs@CC is intrinsically higher than those of other catalysts. The estimation based on the polarization curves indicates that the p-NiCoP/NCFs@CC has a turnover frequency (${\text{TOF}}_{{{\text{HER}}}}$) of 0.367 s^−1^ at the overpotential of 100 mV, which is higher than those of NiCoP/NCFs@CC (0.167 s^−1^), p-NiCoP@CC (0.105 s^−1^), p-CoP/NCFs@CC (0.129 s^−1^), p-NiP/NCFs@CC (0.085 s^−1^), and NCFs@CC (0.117 s^−1^) as shown in Fig. S9a. It well validates that the p-NiCoP/NCFs@CC has the higher intrinsic HER activity.

The electrochemical impedance (EIS) spectra are measured to assess the HER kinetics of the catalysts. Figure S9b shows that the p-NiCoP/NCFs@CC has a lower diameter of the semicircle in the medium-frequency range of the EIS spectra. It indicates that the p-NiCoP/NCFs@CC has a lower charge transfer resistance (*R*_ct_) for the HER. This observation suggests that the HER by the p-NiCoP/NCFs@CC is kinetically faster than those by the NCFs@CC, p-CoP/NCFs@CC, p-NiP/NCFs@CC, NiCoP/NCFs@CC, and p-NiCoP@CC. In particular, the faster HER kinetics of the p-NiCoP/NCFs@CC can be further demonstrated by the Tafel plots in Fig. 4c. It shows that the p-NiCoP/NCFs@CC has a Tafel slope of 68 mV dec^−1^, which is lower than those of p-CoP/NCFs@CC (93 mV dec^−1^), p-NiP/NCFs@CC (112 mV dec^−1^), NiCoP/NCFs@CC (103 mV dec^−1^), p-NiCoP@CC (80 mV dec^−1^), and NCFs@CC (116 mV dec^−1^). Based on the Tafel slope in Fig. 4c, it can be inferred that the HER by the p-NiCoP/NCFs@CC follows the Volmer–Heyrovsky mechanism, i.e., it starts with the adsorption of proton through the discharge of water (Volmer step:$* + {\text{H}}_{2} {\text{O}} + {\text{e}}^{ - } \to *{\text{H}} + {\text{OH}}^{ - }$), followed by the release of H_2_ (Heyrovsky step: $*{\text{H}} + {\text{H}}_{2} {\text{O}} + {\text{e}}^{ - } \to * + {\text{H}}_{2} \uparrow + {\text{OH}}^{ - }$).

The p-NiCoP/NCFs@CC is also efficient for the OER. The polarization curves in Fig. 4d show that the p-NiCoP/NCFs@CC only needs an overpotential of 306 mV to drive 100 mA cm^−2^ ($\eta_{OER.100}$) when subjected to the OER in the alkaline media. This overpotential is lower than those of the p-CoP/NCFs@CC (338 mV), p-NiP/NCFs@CC (354 mV), NiCoP/NCFs@CC (356 mV), p-NiCoP@CC (378 mV), and the NCFs@CC (392 mV). It indicates that the p-NiCoP/NCFs@CC has the highest OER activity among the catalysts investigated in this work. In particular, the factors, which lead to the high catalytic activity of the p-NiCoP/NCFs@CC toward the HER, such as high BET surface area, Pv· richness, Ni doping, and carbon supporting, can improve the OER activity of the p-NiCoP/NCFs@CC as well. More impressively, the OER activity of the p-NiCoP/NCFs@CC is higher than those of the commercial RuO_2_ (Fig. 4d) and other catalysts reported recently, such as MnCoP/CC [28], Cu_3_P/Ni_2_P@CF [31], and NMCP@NF [32] (Table S2). It gives a strong support that the p-NiCoP/NCFs@CC is an efficient catalyst for the OER in the alkaline media.

The ECSA-normalized polarization curves in Fig. 4e show that the p-NiCoP/NCFs@CC only needs an overpotential of 314 mV to drive 6 mA cm^−2^ (${\eta }_{OER,6}^{ECSA}$=314 mV). This value is lower than those of NiCoP/NCFs@CC (337 mV), p-NiCoP@CC (388 mV), p-CoP/NCFs@CC (338 mV), p-NiP/NCFs@CC (352 mV), and NCFs@CC (397 mV). It signifies that the OER activity of the p-NiCoP/NCFs@CC is intrinsically higher than those of other catalysts. This can be further verified by the higher ${\text{TOF}}_{{{\text{OER}}}}$ of the p-NiCoP/NCFs@CC (0.399 s^−1^) at the overpotential of 300 mV than those of NiCoP/NCFs@CC (0.387 s^−1^), p-NiCoP@CC (0.139 s^−1^), p-CoP/NCFs@CC (0.325 s^−1^), p-NiP/NCFs@CC (0.273 s^−1^), and NCFs@CC (0.116 s^−1^). The p-NiCoP/NCFs@CC is also shown to have a higher OER kinetics. The EIS spectra in Fig. S9d indicate that the p-NiCoP/NCFs@CC exhibits a lower diameter of the semicircle in the medium-frequency range than those of NCFs@CC, p-CoP/NCFs@CC, p-NiP/NCFs@CC, NiCoP/NCFs@CC, and p-NiCoP@CC. It suggests that the p-NiCoP/NCFs@CC has a lower charge transfer resistance for the OER and the OER by the p-NiCoP/NCFs@CC can be proceeded in a kinetically faster way. Figure 4f shows that the p-NiCoP/NCFs@CC has an OER Tafel slope of 97 mV dec^−1^, which is lower than those of p-CoP/NCFs@CC (154 mV dec^−1^), p-NiP/NCFs@CC (138 mV dec^−1^), NiCoP/NCFs@CC (121 mV dec^−1^), p-NiCoP@CC (168 mV dec^−1^), and NCFs@CC (197 mV dec^−1^). The result further validates that the p-NiCoP/NCFs@CC has a higher reaction kinetic for the OER.

Since the p-NiCoP/NCFs@CC is demonstrated to be the efficient catalyst for both the HER and the OER, its stabilities toward the HER and the OER in the alkaline media are tested to examine the possibility for applications in overall water splitting. The chronopotentiometric curves in Fig. 4g, h show that whatever for the HER or the OER, the p-NiCoP/NCFs@CC can maintain its high catalytic efficiencies with no distinguishable activity losses observed for > 12 h. It indicates that the p-NiCoP/NCFs@CC is the stable catalyst for both the HER and the OER. The alteration of the current density has no influence on the catalytic stabilities of the p-NiCoP/NCFs@CC. Figure 4i, j indicates that for both the HER and the OER, the p-NiCoP/NCFs@CC shows the increase of the overpotentials with increase of the current densities. At each current density, the p-NiCoP/NCFs@CC shows the stable overpotentials. In particular, for both the HER and the OER, the overpotentials of the p-NiCoP/NCFs@CC can return back to the initial values when the current densities are resumed from the high to low values.

The high HER and OER stabilities can be attributed to the excellent structural robustness of the p-NiCoP/NCFs@CC. The SEM images in Fig. S10 show that the p-NiCoP/NCFs@CC maintains its original morphological appearance after the HER and the OER. The diffraction peaks corresponding to the (111) and (110) planes of the NCFs and the (200), (220), (310), and (420) planes of the CoP_3_ can be still observed in the XRD diffraction patterns of the p-NiCoP/NCFs@CC after the HER and the OER (Fig. S11a). In particular, the signals corresponding to the Pv· can be still detected on the EPR spectra of the p-NiCoP/NCFs@CC after the HER and the OER (Fig. S11b). The elemental analysis by ICP-OES indicates that the ratio of Co and Ni remains relatively constant before and after the HER (Co:Ni:P after HER is 1:0.45:3.04) and the OER(Co:Ni:P after OER is 1:0.42:2.96). Figure S11d–f gives the XPS spectra of Co 2*p*, Ni 2*p*, and P 2*p* for the p-NiCoP/NCFs@CC after the HER and the OER. It shows that the p-NiCoP/NCFs@CC after the HER exhibits the Co 2*p*, Ni 2*p*, and P 2*p* spectra profiles comparable to that before the HER, in which the peaks corresponding to Co-P and Ni–P can be clearly identified. It well corroborates the excellent stabilities of the p-NiCoP/NCFs@CC during the HER processes. This can be further demonstrated by the HRTEM images of the p-NiCoP/NCFs@CC, where the distinct lattice fringes corresponding to the (200) and (310) planes of the cubic structured CoP_3_ can still be observed (Fig. S12). In contrast to those after the HER, the Co 2*p*, Ni 2*p*, and P 2*p* spectra of the p-NiCoP/NCFs@CC after the OER show the differences from those before the OER. As displayed in Fig. S11d–f, all the Co 2*p*, Ni 2*p*, and P 2*p* spectra of the p-NiCoP/NCFs@CC after the OER show the disappearance of the peaks corresponding to Co–P and Ni–P, while the peaks corresponding to Co^3+^ and Ni^3+^ show obvious increases in the intensities. These observations strongly suggest that the surface of the p-NiCoP NPs in the p-NiCoP/NCFs@CC undergo the oxidation during the OER processes, while the bulk of the p-NiCoP NPs in the p-NiCoP/NCFs@CC remains to that before the OER. This is well consistent with the OER polarization curve of the p-NiCoP/NCFs@CC in Fig. 4d, which shows an obvious peak at ~ 1.38 V, assignable to the oxidation of Co and Ni in the p-NiCoP NPs to Co^3+^ and Ni^3+^. TEM reveals that the p-NiCoP/NCFs after the OER exhibits a thin oxide layer on the surface of the p-NiCoP NPs (Fig. S13), similar to the work reported by Fu et al. [33] Previous work has proposed that the phosphides are pre-catalysts for the OER and they will undergo the surface oxidation in the harsh oxidative OER environments [34]. So, the actual electroactive materials of the phosphides for the OER are oxide layers at the surface [33].

### DFT Calculations

**Fig. 5:**
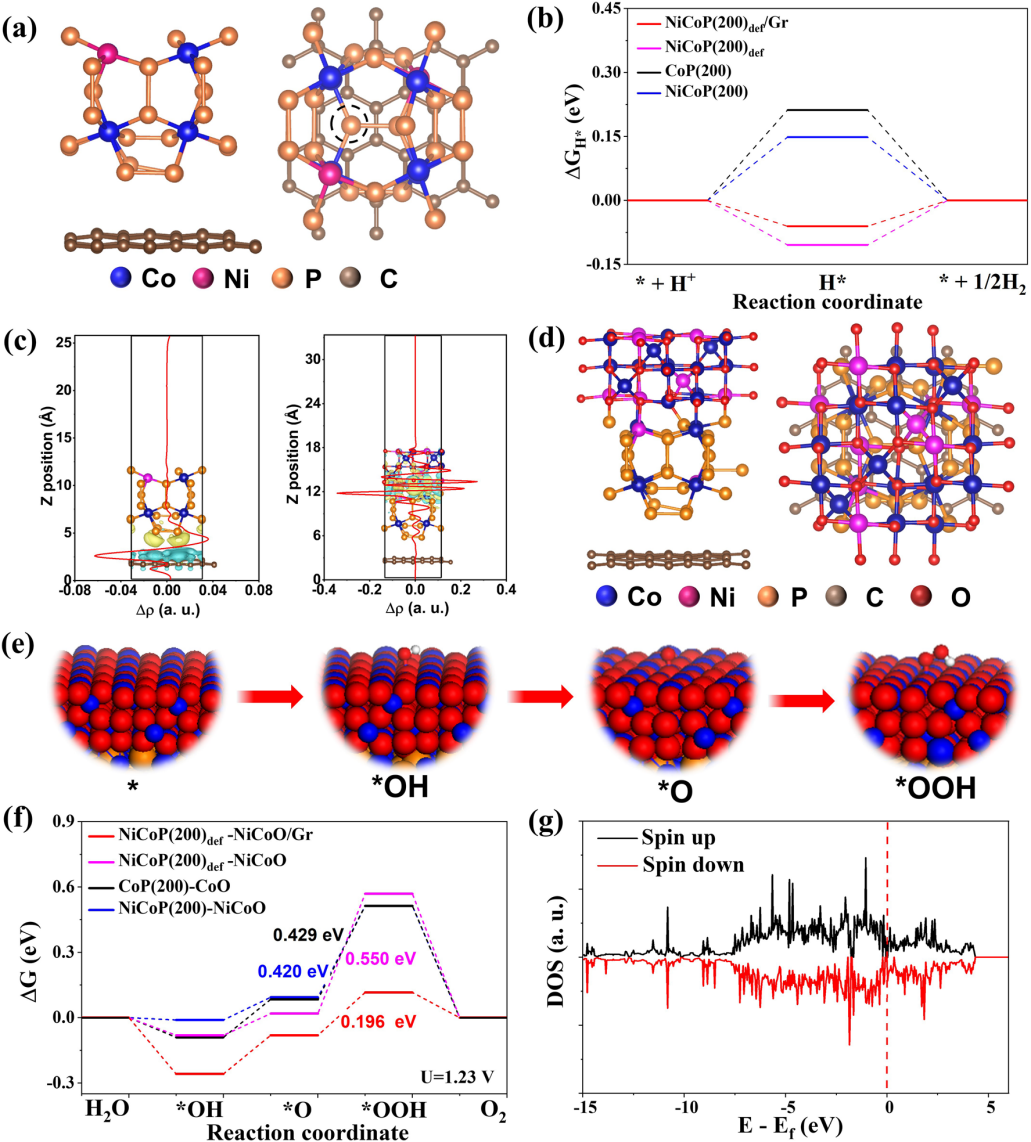
**a** Side and top views of NiCoP(200)_def_/Gr. The red cycle indicates the Pv. **b** Calculated Gibbs free energy of H adsorption. **c** Charge differential analysis of NiCoP(200)_def_/Gr (left) and NiCoP(200)_def_-NiCoO/Gr (right). **d** Side and top views of NiCoP(200)_def_-NiCoO/Gr. **e** Schematic illustration of the OER processes on NiCoP(200)_def_-NiCoO/Gr. **f** Free energy diagram of the OER. **g** DOS spectrum of the NiCoP(200)_def_-NiCoO/Gr

The DFT calculation is conducted to gain deeper understanding on why the Pv· richness, the Ni doping, and the carbon supporting improve the HER/OER catalytic activities of the p-NiCoP/NCFs [35]. Specifically, a cubic structure of Ni-doped CoP_3_ is established for the DFT calculation. Based on the HRTEM images in Fig. 1e, f, the (200) surface of the Ni-doped CoP_3_ (NiCoP(200)) is used to evaluate the catalytic activity of the p-NiCoP/NCFs. The Pv· structure is achieved by the removal of the P atoms from the NiCoP(200). Since the N content in the NCFs is low, the graphene-supported Pv· structure NiCoP(200) (NiCoP(200)_def_/Gr) is directly employed to calculate the influence of the carbon supporting on the catalytic activity of the NiCoP(200)_def_ (Fig. 5a). For the HER, the Gibbs free energy of the H adsorption (ΔG_H*_) has been widely used to evaluate the activity of the catalyst [36]. The active sites for the HER are shown to be the P atoms at the surface of the NiCoP(200)_def_/Gr close to the Pv· (Fig. S14). The Gibbs free energy diagram in Fig. 5b shows that the NiCoP(200)_def_/Gr exhibits a |ΔG_H*_| value of 0.06 eV. This value is closer to 0 eV than that of the CoP_3_(200) (0.21 eV), the NiCoP(200) (0.15 eV), and the NiCoP(200)_def_ (0.11 eV). This observation indicates that the factors, including the Ni doping, the Pv· and the carbon supporting, can all improve the HER catalytic activity of the p-NiCoP/NCFs. It well explains why the p-NiCoP/NCFs@CC exhibits the higher HER activity than the p-CoP/NCFs@CC, the NiCoP/NCFs@CC, and the p-NiCoP@CC, as experimentally shown in Fig. 4a.

The charge analysis is performed to gain better understanding on how the Pv· richness, the Ni doping, and the carbon supporting improve the HER catalytic activity of the NiCoP(200)_def_/Gr. The result indicates that the average oxidation states of Ni, Co and P in the NiCoP(200) are + 0.038|e^−^|, + 0.055|e^−^| and − 0.014|e^−^|, respectively. This is different from the NiCoP(200)_def_, whose average oxidation states of Ni, Co, and P are + 0.051|e^−^|, + 0.082|e^−^| and − 0.022|e^−^|, respectively. It indicates that the presence of the Pv· will increase the oxidation states of Ni and Co bonded to P, while decrease the oxidation state of P bonded to Co/Ni. This finding is in good agreement with the XPS spectra in Fig. 2b–d, which show that the peaks corresponding to Ni and Co bonded to P appear at the relatively higher binding energies, while the peaks corresponding to P bonded to Ni/Co appear at the relatively lower binding energy. This change of the oxidation states alters the adsorption of hydrogen atoms at the surface of the catalysts and subsequently the release of H_2_, which increases the catalytic activity of the NiCoP(200)_def_ toward the HER. The Ni doping can influence the oxidation states of P in the NiCoP(200)_def_ as well. The DFT calculation shows that the oxidation state of P in the CoP(200) is − 0.012|e^−^|, which is higher than that in the NiCoP(200). It indicates that the Ni doping will decrease the oxidation states of P, making it readily adsorption of the H atoms. The incorporation of graphene layer will lead to a strong electronic coupling between NiCoP(200)_def_ and graphene. The charge density difference analysis in Fig. 5c (left) shows that upon the incorporation of graphene layer, the transfer of charges from graphene to NiCoP(200)_def_ occurs. This transfer of charge slightly decreases the adsorption capability of the NiCoP(200)_def_ toward the H atoms, facilitating an easier release of hydrogen from the catalysts, as shown in Fig. 5b. These results indicate that the high HER activity of the p-NiCoP/NCFs@CC mainly arise from its specific structural features of the Pv· richness, the Ni doping, and the carbon supporting.

The OER activity of the p-NiCoP/NCFs is evaluated by the NiCoP(200)_def_/Gr covered with a Ni-doped Co_3_O_4_ layer (the NiCoP(200)_def_-NiCoO/Gr, Fig. 5d). That is because the p-NiCoP NPs on the p-NiCoP/NCFs@CC undergoes the surface oxidation during the OER process, while their bulk remains as the p-NiCoP, as experimentally evidenced by the structural characterization of the p-NiCoP/NCFs@CC after the OER. The actual materials responsible for the OER are the oxide layers at the surface of the p-NiCoP NPs, as mentioned above. The electrochemical OER process mainly involves four elementary steps [37]. It includes the adsorption of OH at the catalyst surface to form *OH, the deprotonation of *OH to form *O, the formation of *OOH through the hydroxylation of *O, and the release of O_2_ through the deprotonation of *OOH (Fig. 5e). The Co atoms at the surface of the NiCoP(200)_def_-NiCoO/Gr is calculated to be the active sites for the OER. The Gibbs free energy diagram in Fig. 5f indicates that the rate determining step (RDS) of the OER by the NiCoP(200)_def_-NiCoO/Gr is the hydroxylation of *O to form *OOH, which has an energy barrier of 0.196 eV. In particular, this energy barrier is much lower than those of NiCoP(200)-NiCoO (0.420 eV), NiCoP(200)_def_-NiCoO (0.550 eV), and CoP(200)-CoO (0.429 eV), which take the hydroxylation of *O to form *OOH as the RDS of the OER as well. This result is consistent with those observed experimentally in Fig. 4d, which shows that the p-NiCoP/NCFs@CC exhibits the higher OER activity than the p-CoP/NCFs@CC, the NiCoP/NCFs@CC, and the p-NiCoP@CC.

The charge difference analysis indicates that there exists a strong electronic coupling between the phosphide nanocatalysts and the surface oxide layer formed during the OER process [38]. The charges are transferred from the phosphide nanocatalysts to the surface oxide layer (Fig. 5c right). With the presence of the Pv·, the Ni doping and the graphene layer, the electronic coupling between the phosphide nanocatalysts and the surface oxide layer will be strengthened. Especially, in the case of the NiCoP(200)_def_-NiCoO/Gr, the transfer of the charges from Gr to NiCoP(200)_def_ would facilitate the transfer of more charges from the phosphide nanocatalysts to the surface oxide layer. This transfer of charges promotes the adsorption of OH^−^ on the catalyst surface, facilitating the subsequent oxidation of OH^−^ to release the O_2_ molecules. Figure 5g shows the density of state (DOS) spectrum of the NiCoP(200)_def_-NiCoO/Gr. It indicates a nonzero DOS at the Fermi level. This observation indicates that the NiCoP(200)_def_-NiCoO/Gr has the higher electric conductivity and can facilitate better electron transfer during the catalytic process. This gives additional evidence that the p-NiCoP/NCFs@CC can exhibit the high activity of the OER. Since the active sites for the OER are the Co atoms at the catalyst surface, we have calculated the Co d-band centers (*ε*_d_) of the various catalysts. Figure S15 indicates that the NiCoP(200)_def_-NiCoO/Gr has a *ε*_d_ value of − 1.43 eV, which is closer to the Fermi level than those of the NiCoP(200)-NiCoO (*ε*_d_ =  − 1.58 eV), the NiCoP(200)_def_-NiCoO (*ε*_d_ =  − 1.51 eV), and the CoP(200)-CoO (*ε*_d_ =  − 1.49 eV). With the d-band center closer to the Fermi level, the adsorption of OH^−^ at the surface of the NiCoP(200)_def_-NiCoO/Gr will be eased (Fig. 5f), which is conducive to the transformation of *OH into *O and *OOH for the subsequent release of O_2_. The result well explains why the p-NiCoP/NCFs@CC exhibits the higher OER activity than the p-CoP/NCFs@CC, the NiCoP/NCFs@CC, and the p-NiCoP@CC. In particular, this observation further indicates that the specific structural features of the Pv· richness, the Ni doping, and the carbon supporting can improve the OER activity of the p-NiCoP/NCFs@CC.

### Overall Water Splitting

**Fig. 6:**
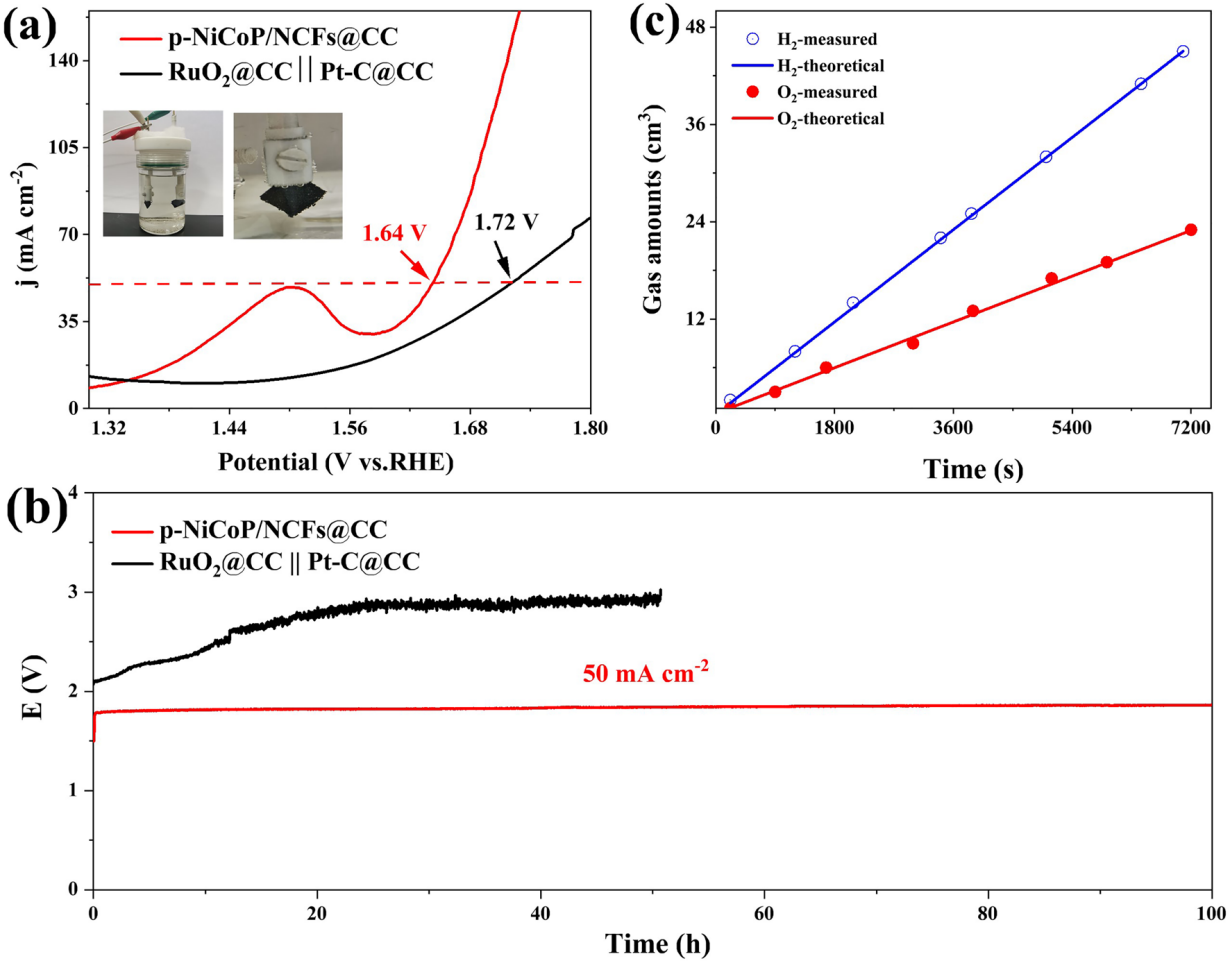
**a** Polarization curves, and **b** chronopotentiometric curve of p-NiCoP/NCFs@CC||p-NiCoP/NCFs@CC and RuO_2_@CC||Pt-C@CC, **c** time-dependent H_2_ and O_2_ amounts generated by the p-NiCoP/NCFs@CC||p-NiCoP/NCFs@CC

Overall water splitting is performed to directly examine the practical usability of the p-NiCoP/NCFs@CC for overall water splitting. Figure 6a shows that the water electrolyzers assembled using the p-NiCoP/NCFs@CC as both the anode and cathode catalysts (p-NiCoP/NCFs@CC||p-NiCoP/NCFs@CC) only need an input voltage of ~ 1.64 V to drive a current density of 50 mA cm^−2^. This input voltage is lower than those of the Pt-C@CC||RuO_2_@CC (~ 1.72 V) and other water electrolyzers reported recently, such as MnCoP/CC [28], CeO_2_-NiCoP_x_/NCF [30], and NiFeCuP [29] (Table S3). It strongly indicates the great potential of using the p-NiCoP/NCFs@CC as the anode and cathode catalyst for water electrolysis. The long-stability measurements show that the p-NiCoP/NCFs@CC||p-NiCoP/NCFs@CC can remain its high performance for overall water splitting with no obvious increase in the input voltage observed to maintain the current density of 50 mA cm^−2^ for > 100 h (Fig. 6b). This is greatly contrast to the Pt-C@CC||RuO_2_@CC, in which the continuous obvious increase of the input voltage can be observed to maintain the current density of 50 mA cm^−2^ with a short period of 20 h. It indicates that the p-NiCoP/NCFs@CC||p-NiCoP/NCFs@CC has a high durability for overall water splitting. The volumes of H_2_ and O_2_ generated during the overall water splitting are determined by a water-drainage method as shown in Fig. 6c. The result indicates that ~ 45 mL of H_2_ and ~ 22 mL of O_2_ can be generated within 120 min under an applied current of 20 mA cm^−2^. Notably, the generated H_2_ and O_2_ amounts nearly meet the theoretical values. The estimation indicates that the Faraday efficiency of water splitting by the p-NiCoP/NCFs@CC||p-NiCoP/NCFs@CC remains at > 99.0% over the time period covered in this work. It indicates a high energy utilization of water electrolysis by the p-NiCoP/NCFs@CC||p-NiCoP/NCFs@CC.

## Conclusions

In summary, the p-NiCoP/NCFs@CC consisting of the p-NiCoP NPs supported in the NCFs has been successfully synthesized by the plasma-assisted phosphatization of the NiCoLDH/NCFs@CC. The p-NiCoP/NCFs@CC shows high activities and excellent stabilities for the HER and the OER in alkaline media. It only needs overpotential of 107 and 306 mV to drive 100 mA cm^−2^ current densities of the HER and OER, respectively. The catalytic activity of the p-NiCoP/NCFs@C are mainly originated from its specific structure of the p-NiCoP NPs, which are rich with the Pv· and has the Ni doping and the carbon supporting. The DFT calculation indicates that the Pv· richness, the Ni doping, and the carbon supporting can optimize the adsorption of the H atoms on the active sites at the catalyst surface, giving the p-NiCoP/NCFs@CC with the high activity for the HER. Additionally, the Pv· richness, the Ni doping, and the carbon supporting can promote the strong electronic couplings between the NCF-supported p-NiCoP NPs with the surface oxide layer formed during the OER process, giving the p-NiCoP/NCFs@CC with the high activity for the OER. When used in the alkaline water electrolyzer, the p-NiCoP/NCFs@CC shows the superior activity and excellent stability for overall water splitting. The work present here is therefore of great interest since the approach reported in this work is extendable to the synthesis of other Pv· richness phosphide NPs with high performance for the catalytic applications.

## Supplementary Information

Below is the link to the electronic supplementary material.

Supplementary file1 (PDF 3306 kb)
