# Supplementary material for: Defect Engineering and Carbon Supporting to Achieve Ni-Doped CoP3 with High Catalytic Activities for Overall Water Splitting
Source: Nanomicro Lett. 2024 Jul 18;16:250. doi: 10.1007/s40820-024-01471-9 (PMC11258119; doi:10.1007/s40820-024-01471-9)
Supplement: Supplementary file 1 — Supplementary file1 (PDF 3306 kb) [file 40820_2024_1471_MOESM1_ESM.pdf]

Supporting Information for

Defect Engineering and Carbon Supporting to Achieve Ni Doped CoP_3 with High Catalytic Activities for Overall Water Splitting

Daowei Zha[1,2], Ruoxing Wang[1,2], Shijun Tian[1], Zhong-Jie Jiang[2,*], Zejun Xu[1], Chu Qin[1], Xiaoning Tian[3,*] and Zhongqing Jiang[1,*]

[1] Department of Physics, Zhejiang Sci-Tech University, Hangzhou, 310018, P. R. China

[2] Guangzhou Key Laboratory for Surface Chemistry of Energy Materials, Guangdong Engineering and Technology Research Center for Surface Chemistry of Energy Materials, College of Environment and Energy, South China University of Technology, Guangzhou, 510006, P. R. China

[3] Department of Materials and Chemical Engineering, Ningbo University of Technology, Ningbo, 315211, P. R. China

*Corresponding authors. E-mail: eszjiang@scut.edu.cn or zhongjiejiang1978@hotmail.com (Zhong-Jie Jiang); boxertxn@hotmail.com (Xiaoning Tian); zhongqingjiang@zstu.edu.cn (Zhongqing Jiang)

S1 Experimental Section

S1.1 Electrochemical Tests

All electrochemical tests were performed at room temperature on an electrochemical station (CHI 760E, CH Instruments Inc, Shanghai) in a standard three-electrode system. The prepared catalysts, a graphite electrode, and a saturated calomel electrode were used as the working, the counter, and the reference electrodes, respectively. 1.0 M KOH was used as the electrolyte. Before data collection, cyclic voltammetry (CV), scanned at 0.068-0.132 V vs. RHE at a scan rate of 50 mV s^{-1}, was applied to electrochemically activate the catalysts.

Linear sweep voltammetry (LSV) was performed in 1.0 M KOH at 10 mV s^{-1}. Tafel slopes were calculated based on the LSV curves. Electrochemical impedance spectra (EIS) were measured at an overpotential of 100 mV from 0.01 Hz to 100 KHz. All the measured potentials vs. the SCE were converted to vs. the RHE by the equation $E_{RHE} = E_{SCE} + 0.059$ pH + 0.242. All the LSVs reported were iR collected.

ECSA was estimated based on the electrochemical double layer capacitance C_{dl}, determined from the CVs measured at different scan rates in the non-Faraday potential region. In this work, since carbon cloth was used as the substrate, which has a certain capacitance value (as shown in **Fig. S8**), the capacitance of carbon cloth was used as the standard value, and the ECSA of each catalyst was calculated by the following formula:

$$ECSA = \frac{C_{dl}\ of catalyst(mF\ cm^{-2})}{C_{dl}\ of\ carbon\ cloth\ (mF\ cm^{-2})\ \ per\ ECSA\ cm^2} \qquad (S1)$$

Taking the p-NiCoP/NCFs@CC as an example, in the HER, its ECSA could be calculated as:

$$ECSA_{\text{p-NiCoP/NCFs@CC}} = \frac{277\ mF\ cm^{-2}}{9\ mF\ cm^{-2} per\ cm^2_{ECSA}} = \ 30.78\ cm^2_{ECSA} \qquad (S2)$$

The TOF values were calculated according to previous reports [S1, S2]:

$$TOF = j \times \frac{A}{n \times F \times m}\ \ S^{-1} \qquad (S3)$$

where, j is the current density (A cm^{-2}). A is the area of the carbon cloth electrode (cm^2). n is the number of electrons transferred (for OER n = 4 and for HER n = 2). F is the Faraday constant (a value of 96485 C mol^{-1}). m is the number of moles of the effective surface sites that are grown onto the carbon cloth.

The total number of effective surface sites was calculated based on the following equation:

$$\frac{\#\text{ Surface sites}}{cm^2\ geometric\ area} = \frac{\#\text{Surface sites (flat standard)}}{cm^2\ geometric\ area} \times \text{Roughness factor} \quad (S4)$$

here the roughness factor (R_f) can be determined by the C_{dl} from **Fig. S8**, and we assume 60 µF cm^{-2} for a flat electrode provided by previous reports [S3]. The surface sites of 2×10^{15} for the flat standard electrode was used for our calculation according to previous results. Thus, using the formula above, the number of surface active sites for the p-NiCoP/NCFs@CC catalyst is estimated to be 2.15×10^{18} surface sites cm^{-2}. Therefore, the TOF for the p-NiCoP/NCFs@CC catalyst at different overpotentials (η) is calculated.

The amounts of the generated H_2 and O_2 were measured by a drainage method. Specifically, the generated H_2 and O_2 during the water splitting were separately flowed into the drain bottles filled with water. The amounts of the generated H_2 and O_2 can be directly obtained by measuring the volumes of the drained water.

The Faraday efficiency calculation formula:

$$The\ Faraday\ efficiency = \frac{actual\ hydrogen\ or\ oxygen\ production}{current\ *\ time\ *\ 0.01119} * 100\% \quad (S5)$$

S1.2 DFT Calculation

The dipole corrected DFT calculations with considerations of spin polarization were performed in use of the Vienna ab-initio simulation package (VASP) [S4]. The electron–electron exchange correlations were described by the generalized gradient approximation (GGA) in the form of the Perdew-Burke-Ernzerhof (PBE) [S5]. The electron-ion interactions were described using the projector augment wave (PAW) type pseudopotential [S6]. An energy cut-off of 450 eV was employed for plane-wave expansion. The respective convergence criteria for energy and force were 1×10^{-5} eV and 0.02 eV $Å^{-1}$. The Brillouin zone was sampled with the Γ-centered k-point mesh for the cell and slab optimization, which was selected to ensure $a_n \times k_n$ (n = 1, 2, 3) >30 Å (a_n is the lattice parameters of a cell). Calculations of the HER/OER energetics were carried out on the (200) surface of the cubic structure of CoP_3 with some Co atoms replaced by Ni (NiCoP(200)). The NiCoP(200) with Pv· (NiCoP(200)$_{def}$) is achieved by removal of the P atoms from the NiCoP(200). To construct the model of the NiCoP(200)$_{def}$/Gr, a NiCoP(200)$_{def}$ slab is stacked on the graphene substrate directly. The NiCoP(200)$_{def}$-NiCoO/Gr slab was established by adding a layer of Ni doped Co_3O_4 on the surface of the NiCoP(200)$_{def}$/Gr. A vacuum slab of > 15 Å in z-direction was added to avoid the artificial interactions between the periodic images. The hydrogen adsorption free energy was computed using the equation:

$$\Delta G_{H*} = (E_{suface+H} - E_{suface}) - 1/2E_{H_2} + \Delta ZPE - T\Delta S \quad (S6)$$

Where ΔZPE and ΔS are the difference in the zero-point energy and entropy between the adsorbed H atom and the gaseous phase H_2. The OER activities of the catalyst were evaluated based on the models developed by Nørskov et al.[S7] It involves our elementary steps. The Gibbs free energy change (ΔG) of each step were computed by the following equation:

$$\Delta G = \Delta E + \Delta ZPE - T\Delta S + \Delta G_U + \Delta G_{pH} \quad (S7)$$

Where ΔE is the binding energy change of the intermediates, ΔZPE and ΔS are the zero-point and entropy changes of each step. ΔG_U = -eU (U is the applied potential). ΔG_{pH} is the pH value correlation of ΔG: $\Delta G_{pH} = k_B T\ln(10)\times pH$.

S2 Supplementary Figures and Tables

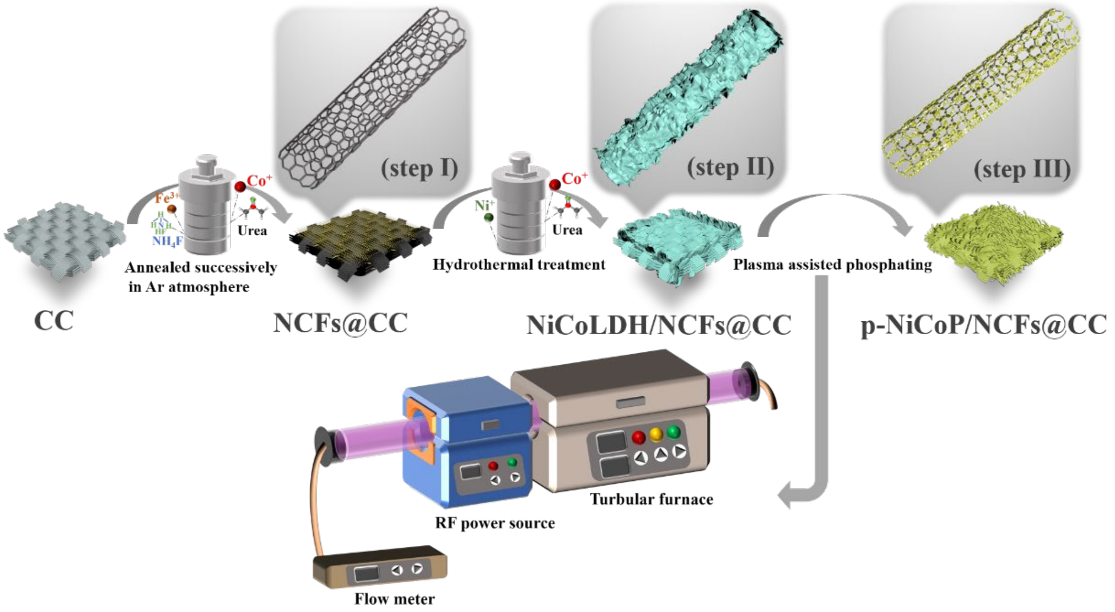

Fig. S1 Illustration of the procedure for the synthesis of the p-NiCoP/NCFs@CC

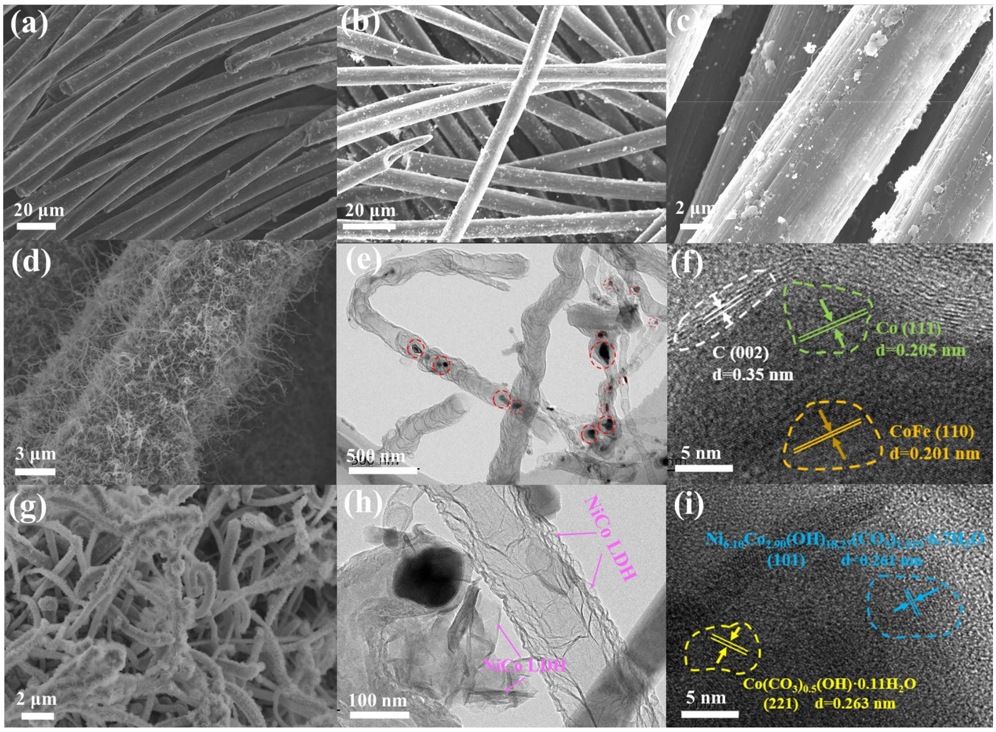

Fig. S2 **a** SEM image of CC, **b, c** SEM images of the CC calcined in the presence of dicyandiamide without the FeCo LDH deposition, **d** SEM image of NCFs@CC, **e** TEM and **f** HRTEM images of NCFs scraped form the NCFs@CC, **g** SEM image of NiCoLDH/NCFs@CC, **h** TEM and **i** HRTEM images of NiCoLDH scraped form the NiCoLDH/NCFs@CC

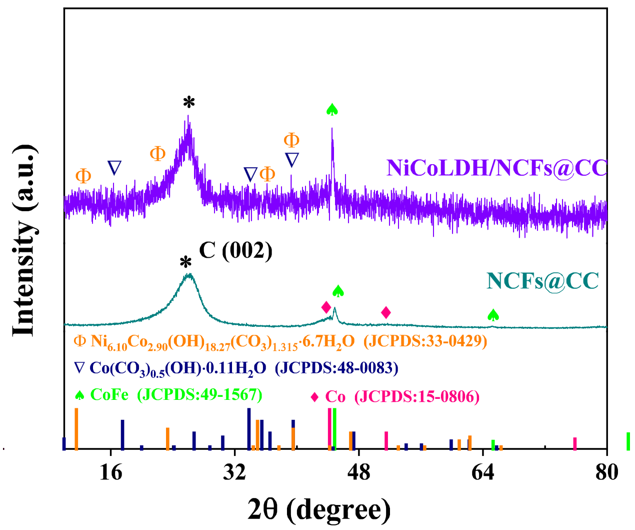

Fig. S3 XRD patterns of NCFs@CC and NiCoLDH@NCFs@CC

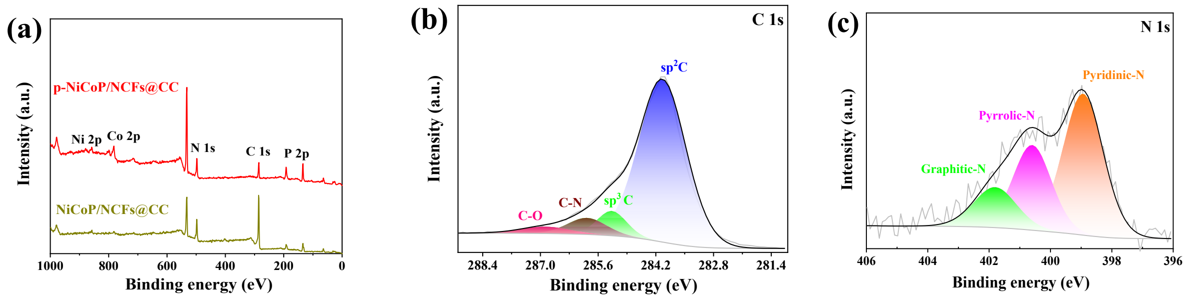

Fig. S4 **a** XPS survey spectrum of p-NiCoP/NCFs@CC. **b** C 1s and **c** N 1s spectra of p-NiCoP/NCFs@CC

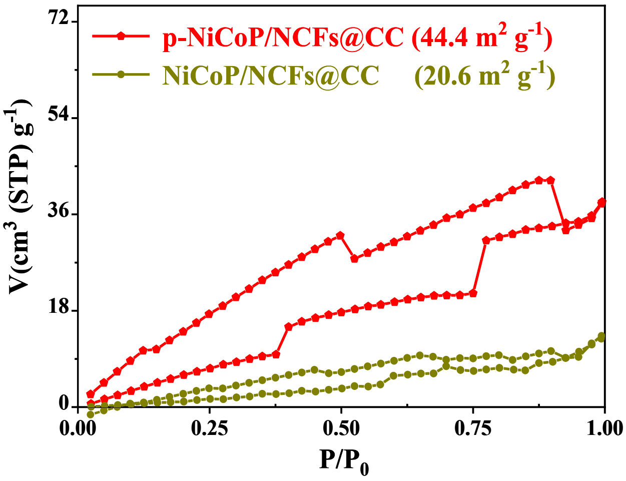

Fig. S5 N_2 adsorption-desorption isotherms

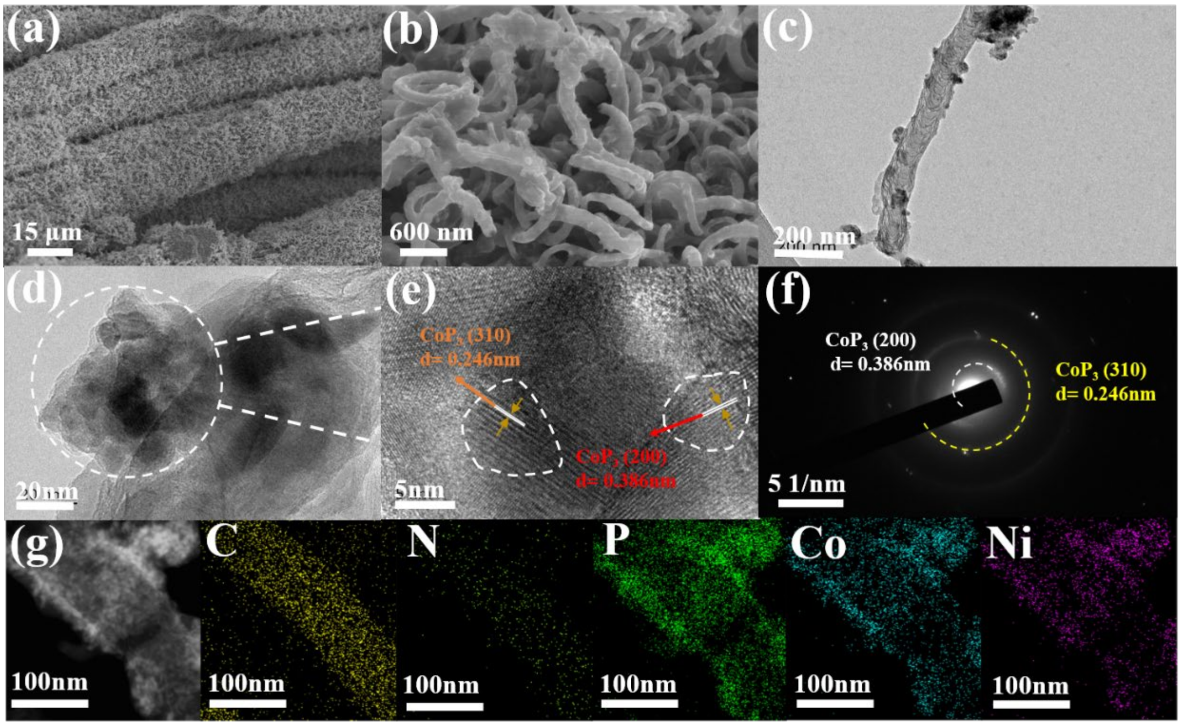

Fig. S6 a, b SEM images of NiCoP/NCFs@CC, **c, d** TEM, **e** HRTEM, **f** SAED pattern, and **g** EDX elemental mapping images of NiCoP/NCFs scraped form the NiCoP/NCFs@CC

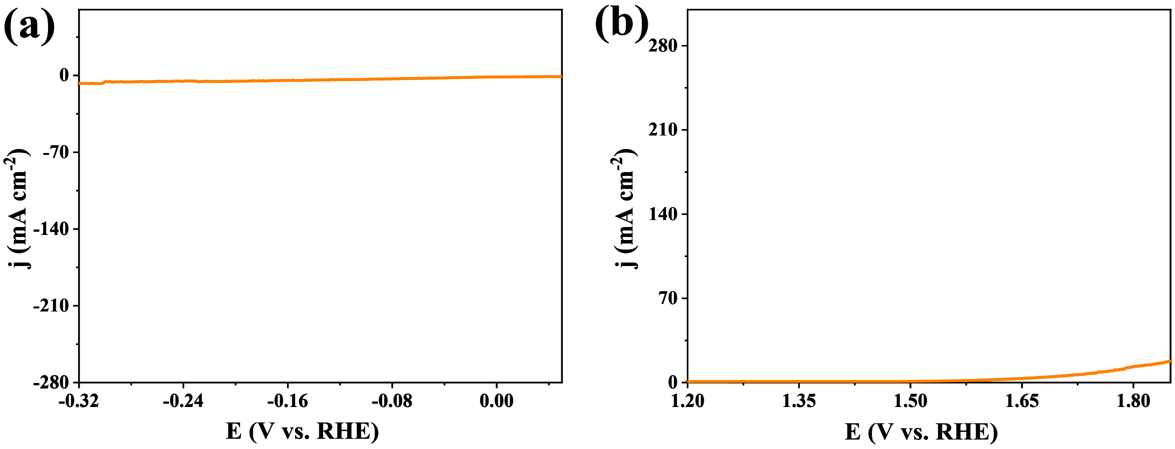

Fig. S7 a HER and **b** OER polarization curves of the CC

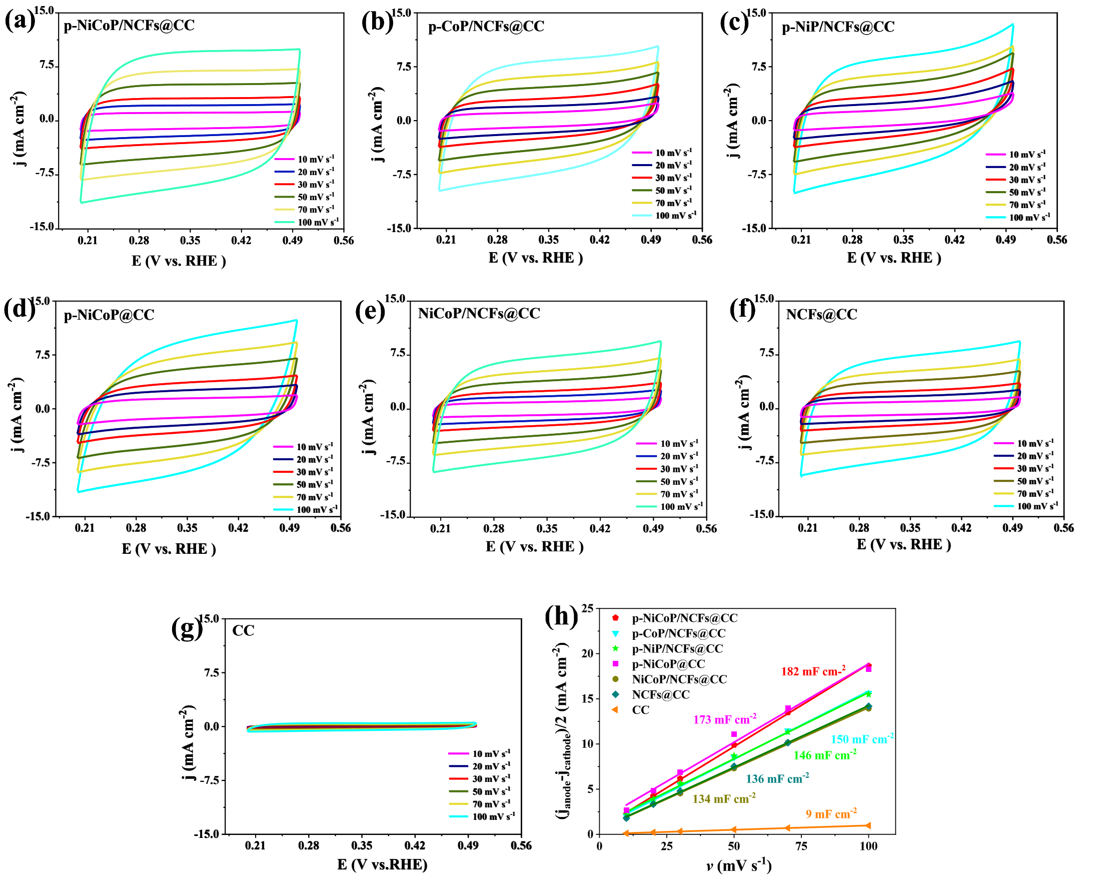

Fig. S8 a-g Electrochemical CVs of the samples at different scanning rates (v), **h** plot of (j_{anode}-$j_{cathode}$)/2 *vs.* scan rate (v), whose slope corresponds to C_{dl} of the catalyst

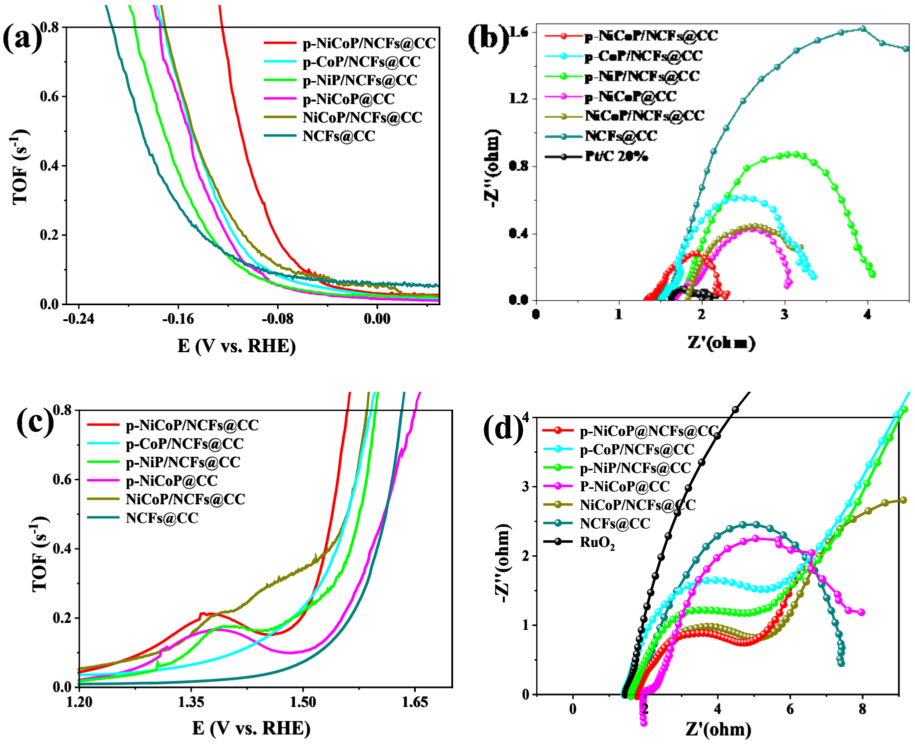

Fig. S9 TOFs of the catalysts for **a** the HER and **c** the OER. EIS spectra of the catalysts for **b** the HER and **d** the OER

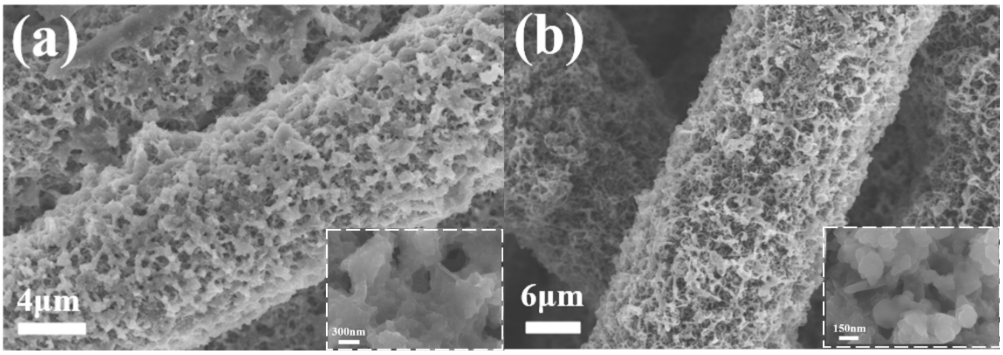

Fig. S10 SEM images of p-NiCoP/NCFs@CC after **a** the HER and **b** the OER.

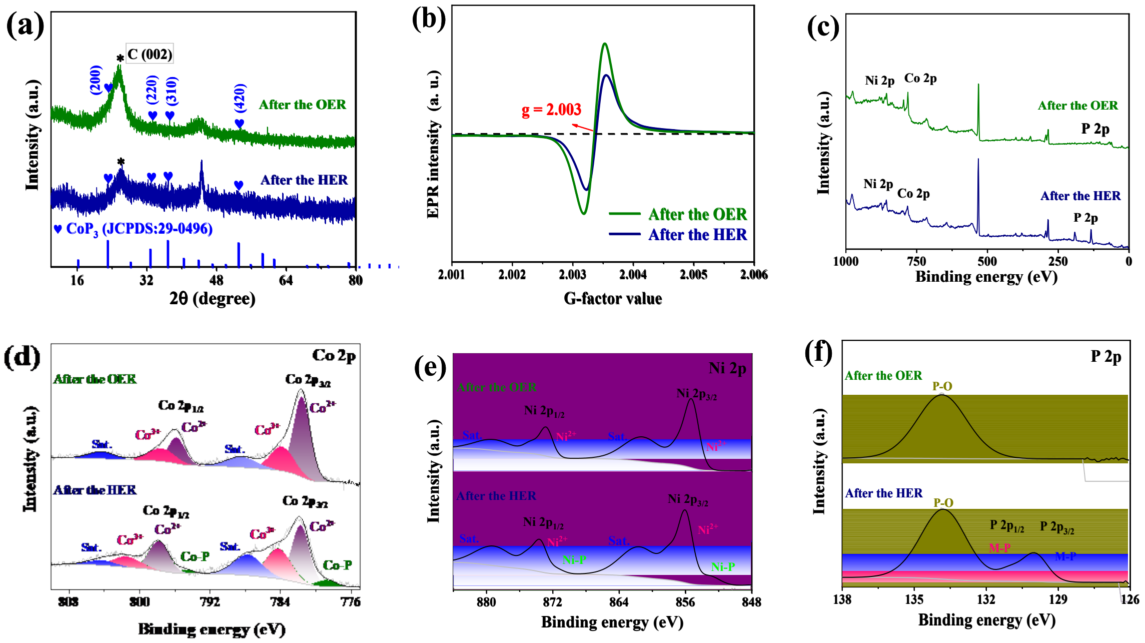

Fig. S11 a XRD pattern, **b** EPR spectra, and **c** XPS survey spectra of the p-NiCoP/NCFs@CC after the HER and the OER. XPS spectra of **d** Co 2p, **e** Ni 2p and **f** P 2p of the p-NiCoP/NCFs@CC after the HER and the OER

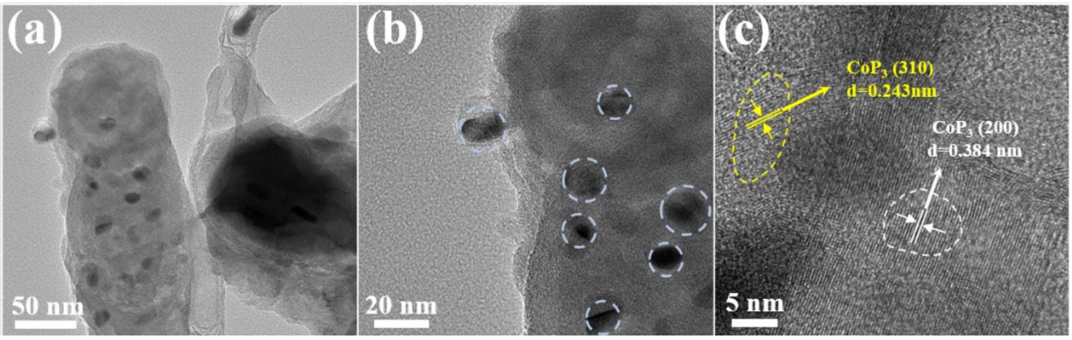

Fig. S12 a TEM, **b, c** HRTEM images of p-NiCoP/NCFs@CC scraped form the p-NiCoP/NCFs@CC after the HER

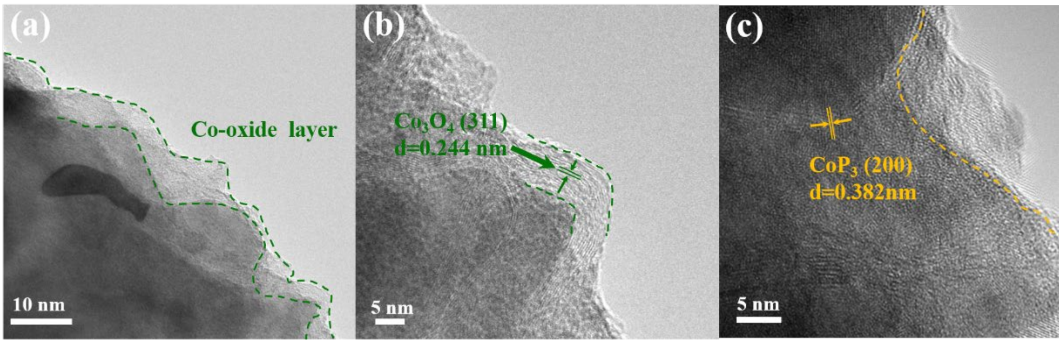

Fig. S13 a TEM and **b, c** HRTEM images of p-NiCoP/NCFs@CC after the OER

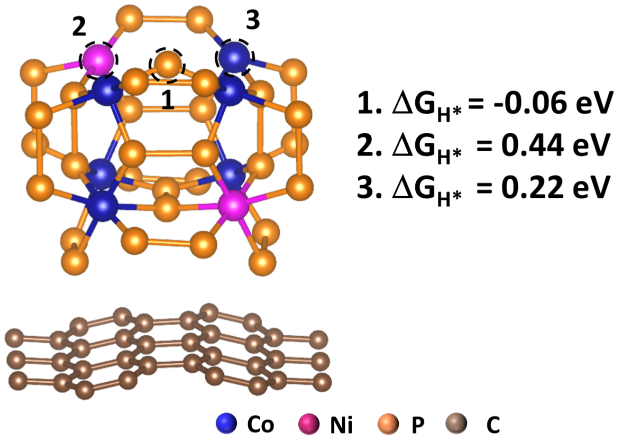

Fig. S14 ΔG_{H*} values of the H adsorption at the different sites on the NiCoP(200)$_{def}$/Gr. It indicates that the P atom close to the Pv· is the active site for the HER since it has a ΔG_{H*} value of -0.06 eV

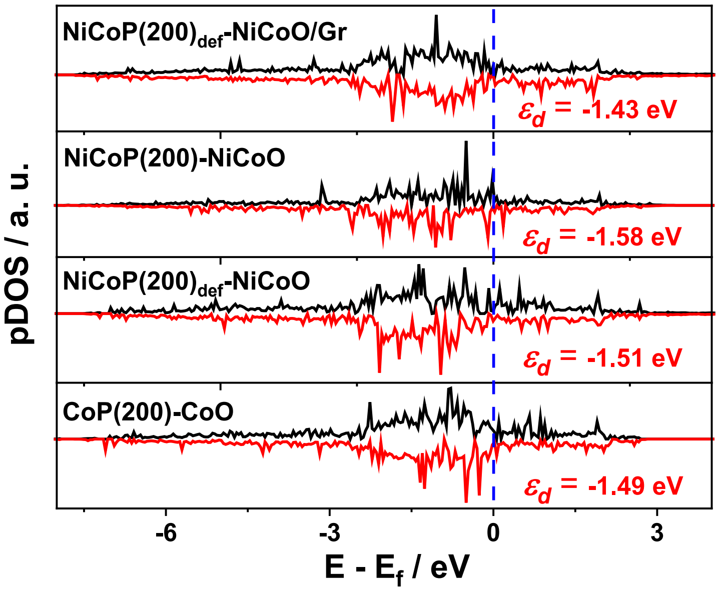

Fig. S15 PDOSs of Co 3d for the NiCoP(200)$_{def}$-NiCoO/Gr, the NiCoP(200)-NiCoO, the NiCoP(200)$_{def}$-NiCoO, and the CoP(200)-CoO. The d-band centers of Co 3d are indicated

Table S1 HER performance comparison of p-NiCoP/NCFs@CC with other TMPs based electrocatalysts

Catalyst	Electrolyte	η(mV)	Tafel slope (mV dec^{-1})	Refs.
**p-NiCoP/NCFs@CC**	**1.0 M KOH**	**η_{10} = 29**<br>**η_{50} = 86**	**68**	**This work**
NMCP@NF	1.0 M KOH	η_{50} = 143	70	[S8]
MnCoP/CC	1.0 M KOH	η_{10} = 69	46.16	[S9]
CeO_2-$NiCoP_x$/NCF	1.0 M KOH	η_{10} = 39	67	[S10]
FN-CoP NS	1.0 M KOH	η_{10} = 66	44.9	[S11]
NiFeCuP	1.0 M KOH	η_{10} = 86	52.3	[S12]
CoFeP NS@NCNF	1.0 M KOH	η_{10} = 113	108	[S13]
NiCoO–2P/S	1.0 M KOH	η_{10} = 143	82	[S14]
Cu_3P/Ni_2P@CF	1.0 M KOH	η_{10} = 88.1	94	[S15]
MoP@N-NiCoP	1.0 M KOH	η_{10} = 43	52	[S16]

Table S2 OER performance comparison of p-NiCoP/NCFs@CC with other TMPs based electrocatalysts

Catalyst	Electrolyte	η(mV)	Tafel slope (mV dec^{-1})	Refs.
**p-NiCoP/NCFs@CC**	**1.0 M KOH**	**η_{50} = 276**<br>**η_{100} =306**	**97**	**This work**
NMCP@NF	1.0 M KOH	η_{50} =280	41	[S8]
MnCoP/CC	1.0 M KOH	η_{100} =460	44.9	[S9]
CeO_2-$NiCoP_x$/NCF	1.0 M KOH	η_{10} =260	72	[S10]
FN-CoP NS	1.0 M KOH	η_{10} = 241	69.6	[S11]
NiFeCuP	1.0 M KOH	η_{10} = 156	21.4	[S12]
CoFeP NS@NCNF	1.0 M KOH	η_{20} = 268	116	[S13]
NiCoO–2P/S	1.0 M KOH	η_{100} = 254	88	[S14]
Cu_3P/Ni_2P@CF	1.0 M KOH	η_{50} =330	72	[S15]
MoP@N-NiCoP	1.0 M KOH	η_{10} = 232	45.85	[S16]

Table S3 Cell performance comparison of the p-NiCoP/NCFs@CC with other water electrolyzers for overall water splitting

Catalyst	Electrolyte	Cell voltage(V) @10 mA cm^{-2}	Refs.
**p-NiCoP/NCFs@CC**	**1.0 M KOH**	**1.325**	**This work**
NMCP@NF	1.0 M KOH	1.52	[S8]
MnCoP/CC	1.0 M KOH	1.68	[S9]
CeO_2-$NiCoP_x$/NCF	1.0 M KOH	1.49	[S10]
FN-CoP NS	1.0 M KOH	1.57	[S11]
NiFeCuP	1.0 M KOH	1.49	[S12]
CoFeP NS@NCNF	1.0 M KOH	1.59	[S13]
NiCoO–2P/S	1.0 M KOH	1.50	[S14]
Cu_3P/Ni_2P@CF	1.0 M KOH	1.56	[S15]
MoP@N-NiCoP	1.0 M KOH	1.54	[S16]

Supplementary References

[S1] J. Jia, L. Li, X. Lian, M. Wu, F. Zheng et al., A mild reduction of Co-doped MnO_2 to create abundant oxygen vacancies and active sites for enhanced oxygen evolution reaction. Nanoscale **13**, 11120-11127 (2021). http://doi.org/10.1039/D1NR02324A

[S2] M. Duraivel, S. Nagappan, K.H. Park, K. Prabakar, Hierarchical 3D flower like cobalt hydroxide as an efficient bifunctional electrocatalyst for water splitting. Electrochim. Acta **411**, 140071 (2022). https://doi.org/10.1016/j.electacta.2022.140071

[S3] H. Zhou, F. Yu, Y. Huang, J. Sun, Z. Zhu et al., Efficient hydrogen evolution by ternary molybdenum sulfoselenide particles on self-standing porous nickel diselenide foam. Nat. Commun. **7**, 12765 (2016). http://doi.org/10.1038/ncomms12765

[S4] G. Kresse, J. Furthmüller, Efficient iterative schemes for ab initio total-energy calculations using a plane-wave basis set. Phys. Rev. B **54**, 11169-11186 (1996). http://doi.org/10.1103/PhysRevB.54.11169

[S5] J.P. Perdew, K. Burke, M. Ernzerhof, Generalized gradient approximation made simple. Phys. Rev. Lett. **77**, 3865-3868 (1996). http://doi.org/10.1103/PhysRevLett.77.3865

[S6] S. Goedecker, M. Teter, J. Hutter, Separable dual-space Gaussian pseudopotentials. Phys. Rev. B **54**, 1703 (1996). https://doi.org/10.1103/PhysRevB.54.1703

[S7] J.K. Nørskov, J. Rossmeisl, A. Logadottir, L. Lindqvist, J.R. Kitchin et al., Origin of the overpotential for oxygen reduction at a fuel-cell cathode. J. Phys. Chem. B **108**, 17886-17892 (2004). https://doi.org/10.1021/jp047349j

[S8] M.R. Kandel, U.N. Pan, D.R. Paudel, P.P. Dhakal, N.H. Kim et al., Hybridized bimetallic phosphides of Ni–Mo, Co–Mo, and Co–Ni in a single ultrathin-3D-nanosheets for efficient HER and OER in alkaline media. Compos. Part B-Eng. **239**, 109992 (2022). https://doi.org/10.1016/j.compositesb.2022.109992

[S9] M. Wang, W. Fu, L. Du, Y. Wei, P. Rao et al., Surface engineering by doping manganese into cobalt phosphide towards highly efficient bifunctional HER and OER electrocatalysis. Appl. Surf. Sci. **515**, 146059 (2020). https://doi.org/10.1016/j.apsusc.2020.146059

[S10] S. Wen, J. Huang, T. Li, W. Chen, G. Chen et al., Multiphase nanosheet-nanowire cerium oxide and nickel-cobalt phosphide for highly-efficient electrocatalytic overall water splitting. Appl. Catal. B: Environ. **316**, 121678 (2022). https://doi.org/10.1016/j.apcatb.2022.121678

[S11] D. Xu, J. Yao, X. Ma, Y. Xiao, C. Zhang et al., F, N neutralizing effect induced Co-P-O cleaving endows CoP nanosheets with superior HER and OER performances. J. Colloid Interf. Sci. **619**, 298-306 (2022). https://doi.org/10.1016/j.jcis.2022.03.123

[S12] J. Ge, S. Diao, J. Jin, Y. Wang, X. Zhao et al., NiFeCu phosphides with surface reconstruction via the topotactic transformation of layered double hydroxides for overall water splitting. Inorg. Chem. Front. **10**, 3515-3524 (2023). http://doi.org/10.1039/D2QI02582E

[S13] B. Wei, G. Xu, J. Hei, L. Zhang, T. Huang et al., CoFeP hierarchical nanoarrays supported on nitrogen-doped carbon nanofiber as efficient electrocatalyst for water splitting. J. Colloid Interf. Sci. **602**, 619-626 (2021). https://doi.org/10.1016/j.jcis.2021.06.045

[S14] F. Yan, L. Yan, X. Wei, Y. Han, H. Huang et al., Structure-design and synthesis of nickel-cobalt oxide/sulfide/phosphide composite nanowire arrays for efficient overall water splitting. Int. J. Hydrogen Energy **47**, 10616-10627 (2022). https://doi.org/10.1016/j.ijhydene.2022.01.108

[S15] H. Liu, J. Gao, X. Xu, Q. Jia, L. Yang et al., Oriented construction Cu_3P and Ni_2P heterojunction to boost overall water splitting. Chem. Eng. J. **448**, 137706 (2022). https://doi.org/10.1016/j.cej.2022.137706

[S16] Y. Zhang, X. Song, X. Guo, X. Li, Design of molybdenum phosphide @ nitrogen-doped nickel-cobalt phosphide heterostructures for boosting electrocatalytic overall water splitting. J. Colloid Interf. Sci. **648**, 585-594 (2023). https://doi.org/10.1016/j.jcis.2023.05.202
